# Native Mass Spectrometry of Membrane Protein–Lipid Interactions in Different Detergent Environments

**DOI:** 10.1021/acs.analchem.4c03312

**Published:** 2024-10-12

**Authors:** Smriti Kumar, Lauren Stover, Lie Wang, Hanieh Bahramimoghaddam, Ming Zhou, David H. Russell, Arthur Laganowsky

**Affiliations:** 1 Department of Chemistry, Texas A&M University, College Station, Texas 77843, United States; 2 Department of Biochemistry and Molecular Biology, Baylor College of Medicine, Houston, Texas 77030, United States

## Abstract

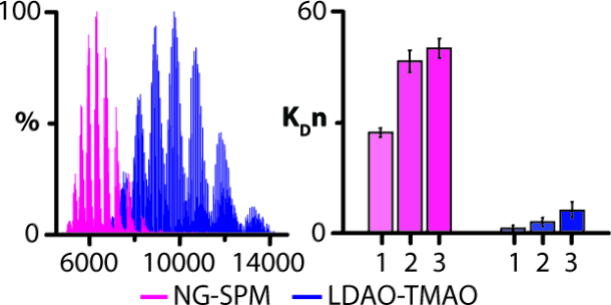

Native mass spectrometry (MS) reveals the role of specific lipids in modulating membrane protein structure and function. Membrane proteins solubilized in detergents are often introduced into the mass spectrometer. However, detergents commonly used for structural studies, such as dodecylmaltoside, tend to generate highly charged ions, leading to protein unfolding, thereby diminishing their utility in characterizing protein–lipid interactions. Thus, there is a critical need to develop approaches to investigate protein–lipid interactions in different detergents. Here, we demonstrate how charge-reducing molecules, such as spermine and trimethylamine-*N*-oxide, enable the opportunity to characterize lipid binding to the bacterial water channel (AqpZ) and ammonia channel (AmtB) in complex with regulatory protein GlnK in different detergent environments. We find that protein–lipid interactions not only are protein-dependent but also can be influenced by the detergent and type of charge-reducing molecule. AqpZ-lipid interactions are enhanced in LDAO (*n*-dodecyl-*N*,*N*-dimethylamine-*N*-oxide), whereas the interaction of AmtB-GlnK with lipids is comparable among different detergents. A fluorescent lipid binding assay also shows detergent dependence for AqpZ-lipid interactions, consistent with results from native MS. Taken together, native MS will play a pivotal role in establishing optimal experimental parameters that will be invaluable for various applications, such as drug discovery as well as biochemical and structural investigations.

## Introduction

Native MS is a widely employed method for investigating the structure and function of biomolecular assemblies.^1−3^ By carefully adjusting the experimental conditions, it can preserve and probe noncovalent interactions, enabling quantitative analysis of binding events and determining stoichiometry of protein complexes.^4−6^ Native MS has proven invaluable in studying biomolecule interactions with small molecules, such as lipids, drugs, and nucleotides, which are essential in biomedical research. Remarkably, examining membrane protein complexes that transport ions and drugs across the cellular membrane has benefited from native MS analysis.^7−9^ Within the membrane environment, lipids have been recognized as critical regulators of membrane protein structure and function.^10−12^

Detergent micelles are typically employed to solubilize membrane proteins for native MS studies.^13^ Minimal collisional energy is applied to release the membrane protein from the detergent micelle.^14^ However, detergents commonly used for structural studies of membrane proteins, such as decylmaltoside (DM) and dodecylmaltoside (DDM), require higher collisional energies to dissociate from the protein.^14^ Consequently, membrane proteins acquire high charge states, resulting in the complex acquiring substantial internal energy. Consequently, the protein experiences significant Coulombic repulsion and tends to undergo unfolding.^14,15^ Charge-reducing detergents, such as C8E4 (tetraethylene glycol monooctyl ether) and LDAO (*n*-dodecyl-*N*,*N*-dimethylamine-*N*-oxide), have been discovered to circumvent this issue.^13,14,16^ Charge-reducing detergents facilitate the production of low-charged ions (with respect to noncharge-reducing detergents), preserving the protein’s native-like structure and noncovalent interactions. Additionally, charge reduction increases the peak spacing, reducing the likelihood of mass spectral peak overlap and improving resolution for higher-order ligand-bound states.^17,18^

Various native MS approaches have been developed to generate charge-reduced ions using nanoelectrospray ionization (nanoESI).^19−21^ One strategy involves the addition of charge-reducing molecules.^17,22^ Trimethylamine-*N*-oxide (TMAO), a natural osmolyte, has been shown to effectively lower the average charge of proteins, enabling the analysis of higher-order lipid-bound states.^18,23,24^ Polyamines, such as spermine (SPM) and spermidine, are more potent and can effectively reduce protein charge at a much lower concentration than TMAO.^25^ SPM-derived detergents have recently been tailored for native MS studies, and they can significantly lower the charge state of membrane protein complexes.^26^ In contrast, other charge-reducing molecules like imidazole have exhibited only minor charge reduction and suffer from significant adduction, leading to poorly resolved mass spectra.^22,27^

An open question is how are membrane protein–lipid interactions influenced in different detergent environments? To this end, we conducted native MS experiments on the bacterial Aquaporin Z (AqpZ) and AmtB-GlnK (a complex between ammonia channel AmtB and soluble regulatory protein GlnK) in various detergent environments: DM (*n*-decyl-ß-maltoside), OGNG (octyl glucose neopentyl glycol), NG (*n*-nonyl-ß-d-glucopyranoside), C8E4 (tetraethylene glycol monooctyl ether), and LDAO (lauryl dimethylamine *N*-oxide). To preserve noncovalent interactions, we used two charge-reducing molecules, SPM and TMAO, which have been shown to reduce charge states for membrane proteins in noncharge-reducing detergents significantly. We systematically characterized the interaction of five different lipids with both membrane proteins in various detergent environments. We also performed lipid titrations in different detergent environments to determine equilibrium dissociation constants (*K*_d_ values) to provide a critical assessment of protein–lipid interactions. These studies are complemented by a fluorescent lipid binding assay.^28^ This work provides critical insight into the impact of detergents on membrane protein–lipid interactions.

## Materials and Methods

### Protein Expression and Purification

Aquaporin Z (AqpZ, UniProt P60844) from *Escherichia coli* containing a C-terminal Strep-tag II (AqpZ-STII), and the AmtB-GlnK complex from *Escherichia coli* was expressed and purified as previously described (see Supporting Information for details).^5,17,29^ The AqpZ-STII expression construct was modified to include a Gly-Cys sequence following the C-terminal Strep-tag II sequence (AqpZ-STII-GC). The protein was expressed and purified as that for AqpZ-STII. The only exception was that the Strep loading buffer was modified to include a wash step with buffer containing 1 mM dithiothreitol (DTT) to reduce cysteines, followed by re-equilibration in the buffer not containing reductant. The eluted protein was mixed with DDM to a final concentration of 0.1% and mixed with a 10-fold excess of Cy3Maleimide (Click Chemistry Tools, stock dissolved in DMSO). The labeling reaction proceeded for 2 h at room temperature. The labeling reaction was quenched by the addition of 1 mM DTT, and DDM was added to a final concentration of 0.5% prior to loading onto a HiPrep 26/10 Desalting column (Cytiva) pre-equilibrated with SPNHA-DDM buffer (100 mM sodium chloride, 10% glycerol, 0.025% DDM, and 20 mM Tris pH 7.4 at room temperature). Peak fractions containing DDM-solubilized Cy3Maleimide labeled AqpZ-STII-GC were aliquoted, flash-frozen in liquid nitrogen, and stored at −80 °C.

### Detergent Exchange

AqpZ-STII was loaded onto a drip column packed with Streptactin Sepharose agarose (IBA Biosciences) pre-equilibrated with SPNHC-C8E4 buffer (100 mM sodium chloride, 10% glycerol, 0.5% C8E4, and 50 mM Tris pH 7.4 at room temperature). After loading, the column was washed with 10 column volumes (CV) of SPNHC-C8E4, followed by a 10 CV wash of SPNHC-wash buffer (100 mM sodium chloride, 10% glycerol, and 50 mM Tris pH 7.4 at room temperature) supplemented with 2× CMC (critical micelle concentration) of the desired detergent. The protein was eluted with 3 mM d-desthiobiotin in SPNHC-wash buffer containing 2× CMC of the desired detergent. AqpZ-STII-GC was detergent-exchanged similarly except that SPNHC-DDM was used instead of SPNHC-C8E4. The AmtB-GlnK complex was detergent-exchanged using a Superdex 200 Increase 10/300 GL column equilibrated with SPNHC-wash buffer supplemented with 2× CMC of the desired detergent and 1 mM ADP.

### Sample Preparation for Native Mass Spectrometry (MS) Analysis

Purified membrane proteins were buffer exchanged into 200 mM ammonium acetate (pH 7.4 at room temperature adjusted with ammonium hydroxide) supplemented with 2× CMC of the desired detergent using a centrifugal desalting column (Micro Bio-Spin 6 columns, BioRad). For the AmtB-GlnK complex, 100 μM ADP was added to the MS buffer. All the phospholipids, including 18:1 Cy5 Cardiolipin (1,1′,2,2′-tetraoleoyl cardiolipin-*N*-(cyanine 5)), were purchased from Avanti Polar Lipids and prepared as previously described (see Supporting Information Materials and Methods for details).^18,30^ Charge-reducing reagents, SPM, and trimethylamine *N*-oxide (TMAO) were purchased from Alfa Aesar and Cayman Chemical, respectively. All of the detergents were purchased from Glycon Biochemicals. Lipids, charge-reducing reagents, and buffer-exchanged protein were prepared in aqueous ammonium acetate and incubated for 2–5 min. The optimized concentrations of AqpZ, AmtB-GlnK complex, SPM, and TMAO were 1 μM, 2 μM, 5 mM, and 60 mM, respectively. For samples without charge-reducing reagents, the same volume of aqueous ammonium acetate was used instead of charge-reducing reagents as previously described.^18^

### Native Mass Spectrometry (MS)

Samples were loaded into a gold-coated borosilicate nanoelectrospray ionization emitters prepared in-house^13^ at room temperature and introduced into an Exactive Plus EMR Orbitrap mass spectrometer (Thermo Scientific). The instrument was optimized for each sample, and detailed instrument settings can be found in Tables S1 and S3. Native MS spectra were processed using UniDec^31^ with the following settings: *m*/*z* range 2000–20000, charge range 5–30, mass sampling every 1 Da, and peak fwhm of 0.85. An in-house Python script (https://github.com/LaganowskyLab/Laganowsky_Lab_Code) was used to calculate the *K*_d_ for each protein–lipid interactions.^32^ The weighted average states (*Z*_avg_) were computed using UniDec.^31^

### Fluorescence Resonance Energy Transfer (FRET) Lipid Binding Assays

AqpZ-STII-GC labeled with Cy3 served as the donor (530 nm excitation, 580 nm emission) and 18:1 Cy5 Cardiolipin (620 nm excitation, 675 nm emission) as the acceptor. FRET (530 nm excitation, 675 nm emission) measurements and correction factors were calculated as previously described.^33,34^ Protein and lipid were both at a final concentration of 0.5 μM. SPNHC-wash buffer with a high concentration of detergent was mixed to make samples containing 10× CMC of detergent. The total protein, lipid, and buffer volume were 50 μL, and the experiments were performed at room temperature in a black 384-well plate (NUNC). Measurements were recorded on a CLARIOstar microplate reader (BMG LABTECH).

## Results

### AqpZ in Different Detergent Environments

**Figure 1:**
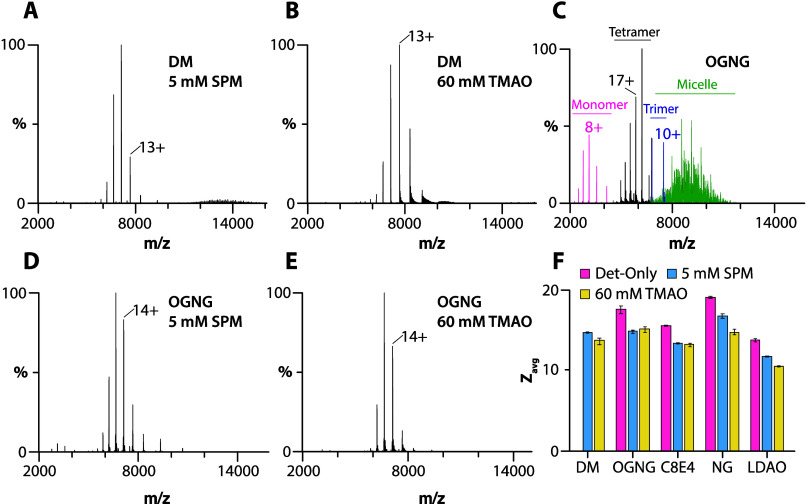
SPM and TMAO preserve the tetramer of AqpZ in different detergent environments. (A, B) Mass spectra of 1 μM AqpZ in DM in the presence of (A) 5 mM SPM and (B) 60 mM TMAO. (C–E) Mass spectra of 1 μM AqpZ in OGNG and in the presence of (D) 5 mM SPM and (E) 60 mM TMAO. (F) The average charge state (*Z*_avg_) of AqpZ in different detergent environments and the presence of charge-reducing molecules. Reported are the mean and standard deviation from three repeated measurements (*n* = 3).

We first investigated AqpZ in different detergent environments (Figures 1, S1, and S2). For tetrameric AqpZ (99.6 kDa) solubilized in DM (2× CMC, 0.174%, ∼3.6 mM), only signals corresponding to those of DM micelles were observed under various instrument settings (Figure S1 and Table S1). In OGNG (2× CMC, 0.116%, ∼2.04 mM), peaks were observed for detergent micelles along with monomer, trimer, and tetramer distributions of AqpZ (Figure 1C). The presence of monomer and trimer signals results from the activation of the complex under these conditions, leading to the dissociation of the intact tetrameric complex. The mass spectrum of the channel in NG (2× CMC, 0.40%, ∼13 mM) had signals for the tetrameric complex, along with some dissociated species (Figure S2C). Despite comparatively mild instrument conditions, signals for dissociated products (monomer and trimer) were also observed but to a lesser extent than those observed for the channel in OGNG (Figures 1C, S2C, and Table S1). The presence of dissociated species presents challenges to studying membrane protein–lipid interactions since conditions that favor subunit dissociation also support the dissociation of noncovalently bound lipids. In contrast, AqpZ in either C8E4 (2× CMC, 0.5%, ∼16 mM) or LDAO (2× CMC, 0.046%, ∼2–4 mM), both of which are charge-reducing detergents,^14,16^ showed only signal for the intact tetrameric complex with a reduction (up to four) in the average charge state (Figures S2 and Table S2). These results illustrate how charge-reducing detergents preserve the integrity of noncovalent interactions.

Next, we explored the utility of charge-reducing molecules, such as SPM and TMAO, to preserve the AqpZ complex in various detergents. For the studies presented herein, SPM and TMAO were added to final concentrations of 5 and 60 mM, respectively. These concentrations were found to be optimal in terms of balancing the charge reduction and signal intensity. Adding either of the charge-reducing molecules to AqpZ in DM displayed a well-resolved mass spectrum of the tetrameric complex with an average charge state (*Z*_avg_) of ∼14 (Figure 1A,B,F and Table S2). Likewise, in the case of AqpZ in OGNG and NG, the use of both SPM and TMAO produced lower charged states (*Z*_avg_ ranging from 15 to 16) and enhanced protein complex stability (Figures 1D,E, S2F, S2I, and Table S2). Adding SPM and TMAO to AqpZ in charge-reducing detergents C8E4 and LDAO reduced *Z*_avg_ by approximately three charges, with *Z*_avg_ ranging from 11 to 13 (Figures 1F and S2D,E,G,H). In short, the addition of SPM and TMAO enables the opportunity to characterize AqpZ-lipid interactions in noncharge-reducing detergents.

### AmtB-GlnK in Different Detergent Environments

Analogous experiments were performed for AmtB-GlnK (166.0 kDa) in different detergent environments and instrumental conditions (Figures S3 and S4 and Table S3). Dissociated species of the AmtB-GlnK complex were observed in DM and OGNG (Figure S3). In the case of NG, the intact AmtB-GlnK complex was observed (Figure S4B). In general, the addition of TMAO to AmtB-GlnK in different detergents led to dissociation of the complex (Figures S3B–D). Mass spectra for the intact AmtB-GlnK complex were obtained in most detergents when SPM was present (Figures S4 and Table S4). In several instances, we observed the adduction of charge-reducing molecules and detergents into the protein complex. TMAO formed a significant number of adducts with the protein in OGNG (Figure S3G). These TMAO adducts could be dissociated at higher collision energies, but this also resulted in the dissociation of the complex (Figure S3B–D). Another interesting observation was the case of AmtB-GlnK in C8E4 and TMAO, where the appearance of detergent adducts was noted (Figure S3H). The detergent environments that displayed adduction were omitted from the studies that followed. In the studies we follow, the intact complex was obtained notably in charge-reducing detergents C8E4 and LDAO, even without charge-reducing molecules (Figure S4D,F).

### Cardiolipin–Protein Interactions

**Figure 2:**
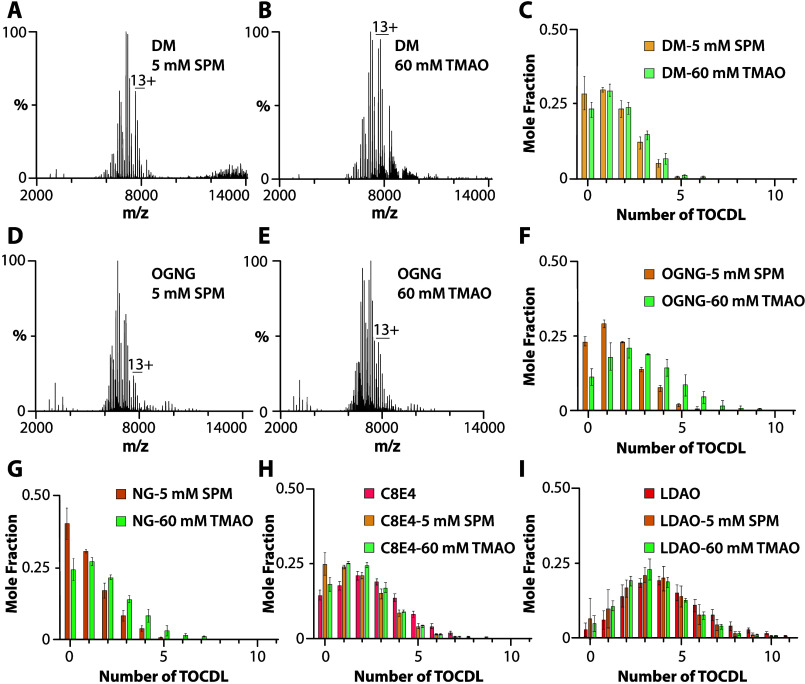
Characterization of AqpZ-TOCDL interactions in different detergent environments. (A, B) AqpZ (1 μM) in DM mixed with 25 μM TOCDL and in the presence of (A) 5 mM SPM and (B) 60 mM TMAO. (C) Mole fraction plot of AqpZ-TOCDL species determined from the deconvolution of the mass spectra shown in (A) and (B). (D–E) AqpZ in OGNG mixed with 25 equiv of TOCDL in the presence of (D) 5 mM SPM and (E) 60 mM TMAO. (F–I) Mole fraction plots for AqpZ and in complex with TOCDL in (F) OGNG, (G) NG, (H) C8E4, and (I) LDAO. Reported are the mean and standard deviation (*n* = 3).

**Figure 3:**
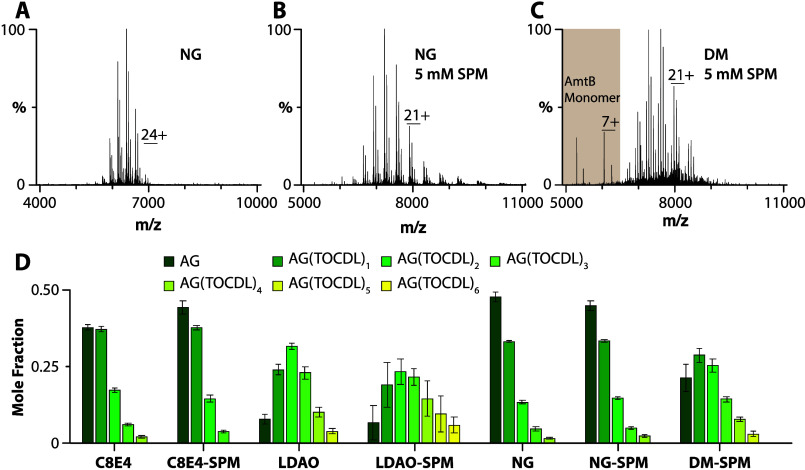
TOCDL binding to AmtB-GlnK(AG) in different detergents. (A, B) Mass spectra of 2 μM AmtB-GlnK mixed with 25 μM TOCDL in NG and in the presence of (B) 5 mM SPM. (C) AmtB-GlnK solubilized in DM with TOCDL and 5 mM SPM. (D) Mole fraction plots of AmtB-GlnK in complex with TOCDL in different detergent environments. Reported are the mean and standard deviation (*n* = 3).

The ability to preserve the intact membrane protein complexes in different detergent environments (described above) inspired us to investigate membrane protein–lipid interactions (Figures 2, 3, and S5). We first focused on cardiolipin, which has been reported to modulate the water transport activity of AqpZ.^16^ The native mass spectrum of AqpZ solubilized in DM with 25 equiv of 18:1 cardiolipin (TOCDL, 1,1′,2,2′-tetraoleoyl-cardiolipin) and in the presence of SPM or TMAO displayed a similar distribution of TOCDL-bound states (Figure 2A–C). In the case of AqpZ solubilized in OGNG or NG, the presence of TMAO gave rise to an increase in the number of TOCDL-bound states compared to SPM, despite both conditions displaying a similar Z_avg_ (Figures 2D–G and S5G,H). In the case of charge-reducing detergents, the addition of SPM or TMAO did not significantly influence the number of TOCDL molecules bound to AqpZ (Figures 2H,I and S5A–F). For the AmtB-GlnK complex (2 μM) with TOCDL (25 μM) in different detergent environments, binding of TOCDL was more prominent in LDAO and DM (Figure 3C,D). The addition of SPM did not change the abundance of TOCDL bound to AmtB-GlnK in C8E4, LDAO, or NG (Figure 3A,B,D). These results show that the binding of TOCDL to both membrane protein complexes is directly influenced by the detergent environment.

### Phosphatidylethanolamine–Protein Interactions

**Figure 4:**
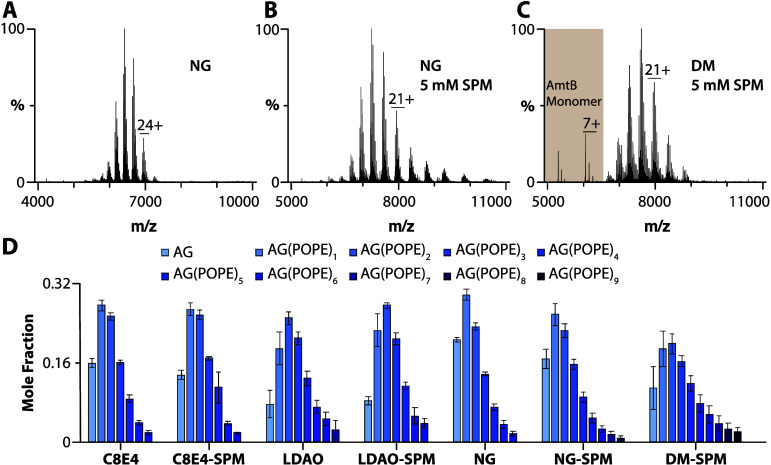
Characterization of POPE binding to AmtB-GlnK in different detergent environments. Mass spectra of 2 μM AmtB-GlnK mixed with 50 μM POPE in (A) NG, (B) NG with 5 mM SPM, and (C) DM with 5 mM SPM. (D) Mole fractions determined from the deconvolution of the mass spectra of AmtB-GlnK with 50 μM POPE in different environments. Reported are the mean and standard deviation (*n* = 3).

We next investigated the binding of 1-palmitoyl-2-oleyl phosphatidylethanolamine (POPE, 16:0 to 18:1), a zwitterionic phospholipid, to AqpZ and AmtB-GlnK in different detergent environments (Figures 4 and S6). Similar to the experiments for TOCDL, the membrane protein and POPE concentrations were held at a fixed concentration. In DM, up to ten POPE molecules bound to AqpZ with similar abundances were observed in the presence of either SPM or TMAO (Figure S6A,B,M). In the case of AqpZ solubilized in OGNG and NG, the addition of SPM or TMAO resulted in a similar number of POPE molecules bound to the membrane protein complex (Figure S6C–F,N–O). However, the mole fraction of higher POPE-bound states of AqpZ was pronounced for the detergent conditions containing TMAO. In C8E4, up to ten POPE molecules bound to AqpZ were observed, and this detergent with TMAO slightly skewed the abundance for a subset of AqpZ-POPE stoichiometries (Figure S6G,H,K,P). Interestingly, AqpZ in LDAO bound up to 17 POPE molecules and was independent of the presence or absence of charge-reducing molecules (Figure S6I,J,L,Q). The variation in the POPE bound states of AmtB-GlnK in different detergent environments was less pronounced (Figure 4). For LDAO and DM, higher-potential POPE-bound states were more abundant, although the total number of POPE-bound states varied slightly (Figure 4C,D). AmtB-GlnK in DM with SPM bound up to nine POPE molecules (Figure 4D). Adding SPM to NG increased the number of POPE molecules bound to AmtB-GlnK (Figure 4D). Like AqpZ, binding of POPE to AmtB-GlnK was independent of SPM in charge-reducing detergents C8E4 and LDAO (Figure 4D).

### Phosphatidylglycerol–Protein Interactions

The binding of the anionic phospholipid 1-palmitoyl-2-oleyl phosphatidylglycerol (POPG, 16:0 to 18:1) to membrane proteins was also investigated (Figures S7 and S8). AqpZ in DM supplemented with SPM or TMAO showed up to ten POPG molecules binding the channel (Figure S7A,B,M). Unlike the other lipids, the presence of TMAO promoted a higher abundance of AqpZ-POPG bound states in DM compared to the same condition with SPM. Similarly, AqpZ in OGNG promoted binding of POPG to AqpZ in the presence of TMAO over SPM (Figure S7C,D,N). AqpZ solubilized in NG bound up to 8 lipids, and the abundance of the different states between the two charge-reducing molecules was comparable (Figure S7E,F,O). POPG binding to AqpZ in C8E4 was comparable to that in NG and independent of charge-reducing molecules (Figure S7G,H,K,P). Like C8E4, AqpZ-POPG interactions were independent of the charge-reducing molecule, but a significant increase in the level of POPG binding (up to 20) was observed (Figure S7I,J,L,Q). Unlike AqpZ, AmtB-GlnK in different detergent environments showed comparable binding of POPG, and the addition of charge-reducing molecules did not significantly impact the abundance of lipid-bound states (Figure S8).

### Other Lipid–Protein Interactions

The other two lipids found in *E. coli* membrane^35−37^ we investigated were 1-palmitoyl-2-oleyl phosphatidic acid (POPA, 16:0 to 18:1) and 1-palmitoyl-2-oleoyl-*sn*-glycero-3-phospho-l-serine (POPS, 16:0–18:1) (Figures S9–S12). In general, AqpZ showed a comparable number of POPA and POPS bound to the channel in DM, OGNG, and NG (Figures S9 and S10). In most cases, the mole fraction of higher AqpZ-lipid bound states in DM, OGNG, and NG was enhanced in the presence of TMAO. AqpZ solubilized in C8E4 showed a similar number of POPAs bound to the complex that was comparable to the protein in noncharge-reducing detergents and independent of charge-reduction (Figure S9G,H,K,P). In LDAO, AqpZ bound nearly 2-fold the number of POPA molecules (Figure S9I,J,L,Q). An appreciable decrease in the level of binding of POPS to AqpZ in C8E4 was observed (Figure S10G,H,K,P). However, AqpZ in LDAO had a similar number of POPS-bound states to those obtained in noncharge-reducing detergents (Figure S10I,J,L,Q). For both C8E4 and LDAO, introducing charge-reducing molecules resulted in no significant change in the abundance of the AqpZ-lipid bound states. POPA and POPS binding to AmtB-GlnK was broadly comparable for the different detergents (Figures S11 and S12). A notable exception was enhanced lipid binding to AmtB-GlnK in LDAO. In the case of POPA, the addition of SPM to AmtB-GlnK in LDAO led to the dissociation of AmtB-GlnK to AmtB (Figure S13) and a reduction in the mole fraction of the higher order of POPS bound states (Figure S12D).

### Determination of Equilibrium Binding Dissociation Constants

**Figure 5:**
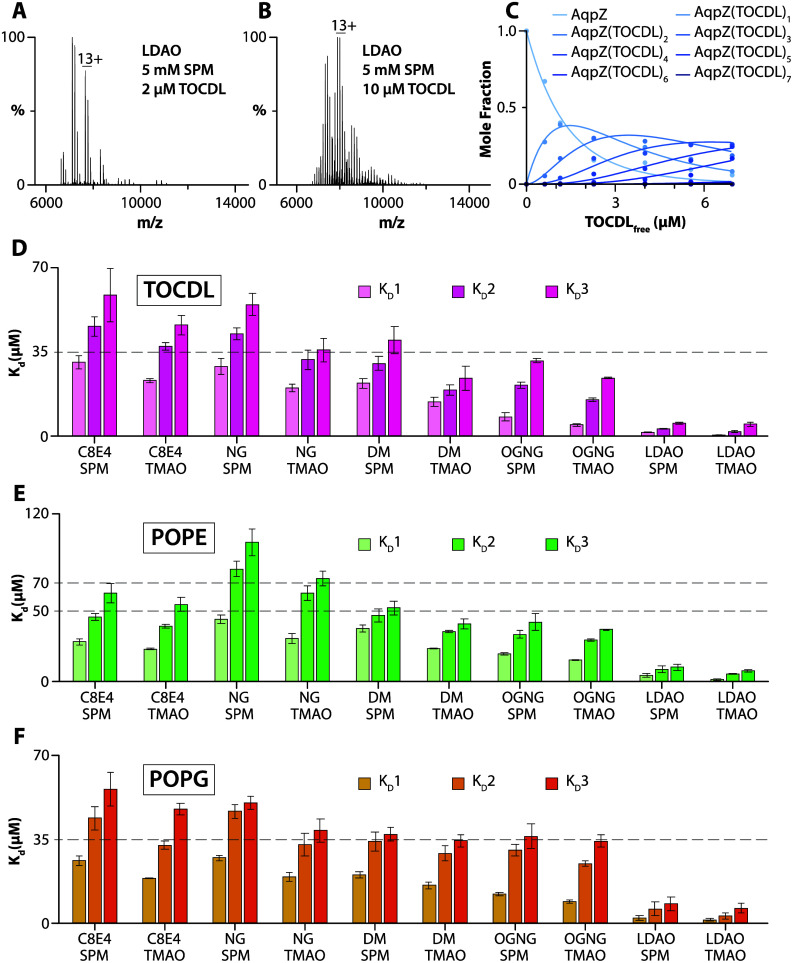
Determination of AqpZ-TOCDL equilibrium dissociation binding constants (*K*_d_ values) in different detergents. (A, B) Representative mass spectra of AqpZ (1 μM) in LDAO mixed with SPM and different concentrations of TOCDL. The concentration of TOCDL is denoted in the inset. (C) Plot of mole fraction data (dots) for AqpZ(TOCDL)_0–7_ determined from a titration series of TOCDL and subsequent fit (*R*^2^ = 0.99) of a sequential lipid binding model (lines). (D–F) Plot of *K*_d_ values for AqpZ binding, (D) TOCDL, (E) POPE, and (F) POPG in different detergent environments. Reported are the mean and standard deviation (*n* = 3).

To gain additional insight into the influence of detergent on protein–lipid interactions, we determined equilibrium dissociation constants (*K*_d_ values) for AqpZ-lipid interactions in different detergents containing either SPM or TMAO (Figures 5 and S14–S42 and Tables S5–S7). For example, mass spectra from a titration series of TOCDL were deconvoluted^31^ to determine the mole fraction of AqpZ(TOCDL)_0–7_ (Figure 5A–C). Subsequently, a sequential lipid binding model was used to determine *K*_d_ values (Figure 5C,D).^32^ Of the different detergent environments, AqpZ displayed the highest binding affinity for TOCDL in LDAO, with the *K*_d_ for binding the first TOCDL (K_d1_) ranging from 0.3 to 1.4 μM (Figure 5D). The binding affinity for TOCDL decreased for AqpZ in the other detergents. The highest *K*_d_ values for TOCDL were observed for AqpZ in C8E4 and NG with SPM. In the case of POPE and POPG, AqpZ in LDAO displayed the highest lipid binding affinity (Figure 5E,F). Like TOCDL, the interactions of POPE and POPG with AqpZ showed a decrease in binding affinity, in which the *K*_d_ values for AqpZ in C8E4 and NG displayed the weakest binding.

### FRET-Based Lipid Binding Assay

**Figure 6:**
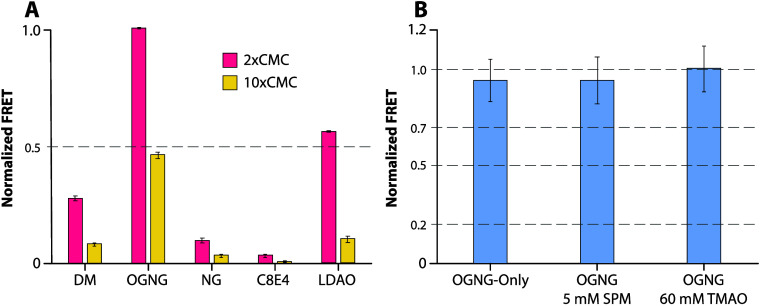
AqpZ binding to TOCDL modified with a fluorophore monitored by FRET. AqpZ modified with cyanine 3 (AqpZCy3) and fluorophore-modified TOCDL (Cy5CDL) were both held at a concentration of 0.5 μM. (A) Plot of FRET for a mixture of AqpZCy3 and Cy5CDL in different detergents at 2 and 10 times the critical micelle concentration (CMC). FRET data are normalized to the largest response. (B) The influence of SPM and TMAO on AqpZCy3 and Cy5CDL interactions. Reported are the mean and standard deviation (*n* = 3).

To complement the native MS studies, we employed a soluble fluorescent lipid binding assay (Figure 6).^28^ In these studies, the binding of TOCDL modified with a cyanine 5 fluorophore (Cy5CDL) to AqpZ labeled with cyanine 3 (AqpZCy3) is monitored by Förster resonance energy transfer (FRET) measurements. An equimolar mixture of Cy5CDL and AqpZCy3 was evaluated in two different concentrations, 2 and 10 times the critical micelle concentration (CMC), of the selected detergent (Figure 6A). The largest FRET signal was observed for the sample in OGNG. In contrast, AqpZ in NG and C8E4 displayed the lowest FRET signals, indicating a reduced level of binding of the fluorophore-modified lipid. In all cases, a 5-fold increase in detergent concentration resulted in a significant reduction in the FRET signal, consistent with the higher concentration of detergent competing with the binding of Cy5CDL to AqpZCy3. We also assessed the impact of SPM and TMAO on lipid binding to AqpZ in OGNG. No statistical difference was observed among the different conditions (Figure 6B).

## Discussion

Membrane proteins are often purified using detergents, and the choice of detergent is usually determined by several criteria, such as the biochemical stability and specific activity of the purified complex. As a result, the selected detergent will be dependent on the target membrane protein complex. Detergents commonly used for structural studies are often not particularly useful for native MS where the goal is to preserve noncovalent interactions.^14,16,38^ Here, mass spectra of AqpZ and AmtB-GlnK in DM, OGNG, and NG illustrate that these environments result in conditions that require considerable collisional activation to liberate the membrane protein from the detergent micelle, which can also promote dissociation of the intact protein complexes. The discovery of charge-reducing detergents,^16,38^ such as C8E4 and LDAO used herein, provide conditions that support mass measurements of intact membrane protein complexes. While these detergents have proven useful for native MS studies, such as those characterizing the binding of lipids and other molecules, a potential problem is that these detergents may not be ideal for purifying various membrane protein complexes that support biochemical stability and activity.

Charge-reducing molecules have been found to be beneficial for promoting the stabilization of membrane proteins and the preservation of noncovalent interactions by promoting the production of ions with lower charge states.^16,19−21,38^ The addition of SPM and TMAO to AqpZ and AmtB-GlnK in different detergents resulted in a reduction of *Z*_avg_ and mass measurements of intact protein complexes. The reduction in *Z*_avg_ depends on each component in the system (Figure S43). The concentrations of SPM and TMAO can be optimized to obtain the desired charge-reduction and signal, as done here. It is worth noting that in some cases we observed the adduction of molecules that are dependent on the protein and detergent. For example, the AmtB-GlnK complex in OGNG with TMAO resulted in a poorly resolved mass spectrum due to the adduction of TMAO and OGNG. Another example is the AmtB-GlnK complex in C8E4 with TMAO, which resulted in the adduction of C8E4. Notably, AqpZ in these detergent environments did not suffer from adducts. We have previously reported that membrane protein solubilized in DDM displayed a broad mass spectrum in the presence of SPM and TMAO. However, the use of SPM-detergents can produce charge-reduced ions of membrane proteins in DDM.^26^ In short, the growing arsenal of charge-reducing molecules affords the opportunity to charge-reduce membrane proteins in different detergent environments.

Native MS reveals that membrane protein–lipid interactions are not only dependent on the protein but also can be influenced by the detergent environment. In the case of AqpZ, LDAO supports an environment that promotes the binding of various lipids. Moreover, lipid binding is largely independent of the addition of SPM and TMAO. On the other hand, AqpZ in the other detergents showed a similar number of lipids bound to the water channel. There are some notable exceptions where the addition of a charge-reducing molecule results in enhanced binding, such as for the binding of POPS to AqpZ in OGNG with TMAO. These results are consistent with the fluorescent lipid binding assay, in which OGNG and LDAO showed the most binding. Most interestingly, the binding of lipids to AmtB-GlnK in the different detergent environments displayed less variation as compared to AqpZ. More specifically, the binding of POPA was comparable for the complex in C8E4, LDAO, and DM. These results illustrate the detergent environment can influence lipid binding.

While the results from experiments using a fixed molar ratio of protein to lipid are illuminating, the determination and evaluation of K_d_s for AqpZ-lipid interactions provide a more quantitative assessment. Interestingly, AqpZ in LDAO displays the highest lipid binding affinity. AqpZ in NG and C8E4 consistently gave the highest *K*_d_ values, implying that these detergents are more effective at competing with AqpZ-lipid interactions. The *K*_d_ values for AqpZ-lipid interactions in different detergents will be a composite of lipid binding to AqpZ and the competition of detergent binding at the specific lipid binding site. Evaluation of the fold change in subsequent *K*_d_ values, for example, *K*_d2_/*K*_d1_, shows that nearly all the detergent environments are comparable (Figure S44) and lipid binding trends are in accordance with previous studies irrespective of the detergent.^39^ In contrast, LDAO, which supported a higher lipid binding affinity to AqpZ, displayed the largest fold change in binding affinity for the binding of subsequent lipids. This result may suggest that LDAO may promote specific binding of the first lipid compared to other detergents.

Detergents are commonly classified into “harsh” and “mild” categories, depending on their capacity to preserve the integrity of membrane structures,^40−44^ which may be the result of removing or preserving essential protein–lipid interactions. Interestingly, LDAO, a zwitterionic detergent considered to be “harsh”, promotes the interaction of AqpZ with various lipids. For example, AqpZ-TOCDL interactions are enhanced by more than 20-fold compared to that in DM, a detergent considered to be “mild”. In contrast, the interaction of lipids with AmtB-GlnK was largely independent of the detergent environment. While the categorization of detergents is largely anecdotal, this report demonstrates how different combinations of detergents with varying CMC (Table S8) and charge-reducing molecules can be exploited for native MS studies of membrane proteins. It is also important to emphasize that these different detergent environments open up an exciting opportunity to study a broader range of membrane proteins, especially those that are not biochemically stable or active in charge-reducing detergents. Taken together, native MS will be instrumental in defining experimental conditions that support the high-affinity binding of ligands, such as lipids and other molecules, to membrane proteins that will be of critical importance for drug discovery and biochemical and structural studies.

## Supporting Information Available

Representative and deconvoluted mass spectra; bar charts of *Z*_avg_ and binding affinities, mole fractions, and corresponding fits with a sequential lipid binding model; tabulated instrument settings, *Z*_avg_ values, binding affinities, and CMCs (PDF)
