# Supplementary material for: Native Mass Spectrometry of Membrane Protein–Lipid Interactions in Different Detergent Environments
Source: Anal Chem. 2024 Oct 12;96(42):16768–76. doi: 10.1021/acs.analchem.4c03312 (PMC11503522; doi:10.1021/acs.analchem.4c03312)
Supplement: Supplementary file 1 — ac4c03312_si_001.pdf [file ac4c03312_si_001.pdf]

Supporting Information

Native mass spectrometry of membrane protein-lipid interactions in different detergent environments

Smriti Kumar,[1] Lauren Stover,[1] Lie Wang,[2] Hanieh Bahramimoghaddam,[1] Ming Zhou,[2] David H. Russell,[1] and Arthur Laganowsky[1*]

[1]Department of Chemistry, Texas A&M University, College Station, Texas 77843, United States

[2]Department of Biochemistry and Molecular Biology, Baylor College of Medicine, Houston, Texas 77030, United States

*Correspondence: ALaganowsky@chem.tamu.edu

Table of Content

- **Materials and Methods**
- **Figure S1** AqpZ in DM
- **Figure S2** AqpZ in C8E4, LDAO, and NG with and without SPM and TMAO
- **Figure S3** AmtB-GlnK in different environments
- **Figure S4** AmtB-GlnK in different environments and Z_{avg}
- **Figure S5** AqpZ binding to TOCDL in different environments
- **Figure S6** AqpZ binding to POPE in different environments
- **Figure S7** AqpZ binding to POPG in different environments
- **Figure S8** AmtB-GlnK with POPG in different environments
- **Figure S9** AqpZ binding to POPA in different environments
- **Figure S10** AqpZ binding to POPS in different environments
- **Figure S11** AmtB-GlnK with POPA in different environments
- **Figure S12** AmtB-GlnK with POPS in different environments
- **Figure S13.** AmtB-GlnK with POPA in LDAO with SPM
- **Figure S14** Mole fraction plot for AqpZ-TOCDL in LDAO with 60 mM TMAO
- **Figure S15** Mole fraction plot for AqpZ-TOCDL in C8E4 with 5 mM SPM
- **Figure S16** Mole fraction plot for AqpZ-TOCDL in C8E4 with 60 mM TMAO
- **Figure S17** Mole fraction plot for AqpZ-TOCDL in NG with 5 mM SPM
- **Figure S18** Mole fraction plot for AqpZ-TOCDL in NG with 60 mM TMAO

- **Figure S19** Mole fraction plot for AqpZ-TOCDL in OGNG with 5 mM SPM
- **Figure S20** Mole fraction plot for AqpZ-TOCDL in OGNG with 60 mM TMAO
- **Figure S21** Mole fraction plot for AqpZ-TOCDL in DM with 5 mM SPM
- **Figure S22** Mole fraction plot for AqpZ-TOCDL in DM with 60 mM TMAO
- **Figure S23** Mole fraction plot for AqpZ-POPE in LDAO with 5 mM SPM
- **Figure S24** Mole fraction plot for AqpZ-POPE in LDAO with 60 mM TMAO
- **Figure S25** Mole fraction plot for AqpZ-POPE in C8E4 with 5 mM SPM
- **Figure S26** Mole fraction plot for AqpZ-POPE in C8E4 with 60 mM TMAO
- **Figure S27** Mole fraction plot for AqpZ-POPE in NG with 5 mM SPM
- **Figure S28** Mole fraction plot for AqpZ-POPE in NG with 60 mM TMAO
- **Figure S29** Mole fraction plot for AqpZ-POPE in OGNG with 5 mM SPM
- **Figure S30** Mole fraction plot for AqpZ-POPE in OGNG with 60 mM TMAO
- **Figure S31** Mole fraction plot for AqpZ-POPE in DM with 5 mM SPM
- **Figure S32** Mole fraction plot for AqpZ-POPE in DM with 60 mM TMAO
- **Figure S33** Mole fraction plot for AqpZ-POPG in LDAO with 5 mM SPM
- **Figure S34** Mole fraction plot for AqpZ-POPG in LDAO with 60 mM TMAO
- **Figure S35** Mole fraction plot for AqpZ-POPG in C8E4 with 5 mM SPM
- **Figure S36** Mole fraction plot for AqpZ-POPG in C8E4 with 60 mM TMAO
- **Figure S37** Mole fraction plot for AqpZ-POPG in NG with 5 mM SPM
- **Figure S38** Mole fraction plot for AqpZ-POPG in NG with 60 mM TMAO
- **Figure S39** Mole fraction plot for AqpZ-POPG in OGNG with 5 mM SPM
- **Figure S40** Mole fraction plot for AqpZ-POPG in OGNG with 60 mM TMAO
- **Figure S41** Mole fraction plot for AqpZ-POPG in DM with 5 mM SPM
- **Figure S42** Mole fraction plot for AqpZ-POPG in DM with 60 mM TMAO
- **Figure S43** Z_{avg} for AqpZ-lipid in different environments
- **Figure S44** Fold change in K_ds in different environments
- **Table S1** Instrument settings for AqpZ
- **Table S2** Z_{avg} of AqpZ in different environments
- **Table S3** Instrument settings for AmtB-GlnK
- **Table S4** Z_{avg} of AmtB-GlnK in different environments
- **Table S5** K_ds for AqpZ-TOCDL
- **Table S6** K_ds for AqpZ-POPE
- **Table S7** K_ds for AqpZ-POPG
- **Table S8** Critical micelle concentration (CMC) of detergents used

Materials and Methods

Expression and Purification of AqpZ. AqpZ containing a C-terminal Strep-tag II (AqpZ-STII) was expressed in *E. coli* BL21-A1 (Invitrogen). A single colony was used to inoculate 100 mL of LB (IBI Scientific) and grown overnight at 37°C while shaking. The overnight culture was used to inoculate TB (IBI Scientific) and allowed to grow at 37°C until the culture reached an OD_{600} of 0.6. Arabinose was added to a final concentration of 0.2%, and the culture was grown overnight at 20°C while shaking. Cells were harvested by centrifugation at 5,000*g* for 10 min, resuspended in lysis buffer (150 mM sodium chloride, 50 mM Tris pH 7.4 at room temperature), lysed with 4-5 passes through an M-110P microfluidizer (Microfluidics) operating at 25,000 PSI, and the lysate clarified by centrifugation at 20,000*g* for 25 min at 4°C. Crude membranes were pelleted by centrifugation for 2 hrs at 100,000*g* at 4°C. The membrane pellet was resuspended in extraction buffer (100 mM sodium chloride, 20% glycerol, 20 mM Tris pH 7.4 at room temperature) and extracted overnight with 5% octyl glucoside (OG, this detergent and others purchased from Glycon Biochemicals GmbH). The supernatant was filtered and loaded onto a StrepTrap HP 5mL column (Cytiva) pre-equilibrated with SPNHA-DDM buffer (100 mM sodium chloride, 10% glycerol, 0.025% DDM, and 20 mM Tris pH 7.4 at room temperature). After loading, the column was washed with 20 mL of SPNHA-DDM supplemented with 2% OG, followed by 25 mL SPNHA-DDM until a steady baseline was reached. AqpZ was eluted with SPNHB-DDM (100 mM sodium chloride, 10% glycerol, 3 mM D-desthiobiotin, 0.025% DDM, and 20 mM Tris pH 7.4 at room temperature), concentrated using a 50 kDa MWCO concentrator (Millipore), and loaded onto a Superdex 200 Increase 10/300 GL column (GE Healthcare) equilibrated with SPNHC-C8E4 buffer (100 mM sodium chloride, 10% glycerol, 0.5% C8E4, and 50 mM Tris pH 7.4 at room temperature). The peak fractions containing C8E4-solubilized AqpZ were aliquoted, flash-frozen in the liquid nitrogen, and stored at -80°C before use.

AmtB-GlnK Complex Purification. GlnK containing an N-terminal HRV3C protease cleavable Strep-tag II and maltose binding protein (STII-TEV-MBP-HRV3C-GlnK) was expressed from pCOLA in *E. coli* BL21(DE3) (New England Biolabs). Protein expression was induced with 0.1 mM IPTG and grown overnight at 20°C. 8×His-HRV3C-AmtB was expressed from pCDF in *E. coli* BL21-A1

(Invitrogen), induced with 0.2% arabinose and grown overnight at 20°C. Cell harvesting, resuspension, and lysis for both proteins were identical to that described for the purification of AqpZ. Post lysis, the lysate containing GlnK was clarified by centrifugation at 40,000*g* for 20 mins, filtered, and loaded onto a 5 mL MBPTrap HP column (Cytiva) equilibrated with MBP-loading buffer (100 mM sodium chloride, 10% glycerol, and 20 mM Tris; pH 7.4 at room temperature). The protein was eluted with MBP-elution buffer (100 mM sodium chloride, 10% glycerol, and 20 mM Tris, 10 mM maltose; pH 7.4 at room temperature) concentrated and loaded onto a HiLoad 16/600 Superdex 200 pg column (GE Healthcare) equilibrated in MBP-loading buffer. Membrane isolation and detergent extraction steps for AmtB were identical to AqpZ's. AmtB was first purified using HisTrap HP 5 mL column (Cytiva) pre-equilibrated with NHA-DDM-1 buffer (200 mM sodium chloride, 10% glycerol, 0.025% DDM, 20 mM imidazole, and 20 mM Tris; pH 7.4 at room temperature). After elution with NHB-DDM buffer (100 mM sodium chloride, 10% glycerol, 0.025% DDM, 500 mM imidazole, and 20 mM Tris; pH 7.4 at room temperature), the pooled sample was loaded onto a HiPrep 26/10 Desalting column pre-equilibrated with NHA-DDM-2 buffer (100 mM sodium chloride, 10% glycerol, 0.025% DDM, 20 mM imidazole, and 20 mM Tris; pH 7.4 at room temperature). Purified tagged AmtB and GlnK were mixed at a molar ratio 1:3 supplemented with 1 mM adenosine diphosphate (ADP) and HRV3C protease (50:1 protein to protease ratio). The mixture was incubated overnight at 4°C and loaded onto a drip column packed with Ni-NTA (IBA Biosciences) pre-equilibrated with NHA-DDM-2 buffer. The flow through was collected and loaded onto a Superdex 200 Increase 10/300 GL column equilibrated with SPNHC-C8E4 buffer supplemented with 1 mM ADP. Peak fractions containing AmtB-GlnK were collected, flash-frozen in liquid nitrogen, and stored at -80°C.

Lipid Preparation for MS analysis. Lipids were prepared as previously described.[1, 2] Purchased lipids were aliquoted, dried under the nitrogen gas flow. They were kept overnight under vacuum to get rid of any trace of organic solvent. The dried lipid films were resuspended in the deionized water to the desired concentrations (~2 mM) and stored at -20°C. For the experiment, small volumes of this stock were pipetted out and diluted with the MS buffer with the desired detergents.

Determination of equilibrium binding constants. Equilibrium binding constants for protein-lipid interactions were determined as previously described.[3, 4] In short, mass spectra from a lipid titration series were deconvoluted using UniDec[5] to determine the abundance of molecular species. A sequential lipid binding model was fit to the experimental data to determine the binding constants for each lipid binding event.

Supporting Figures

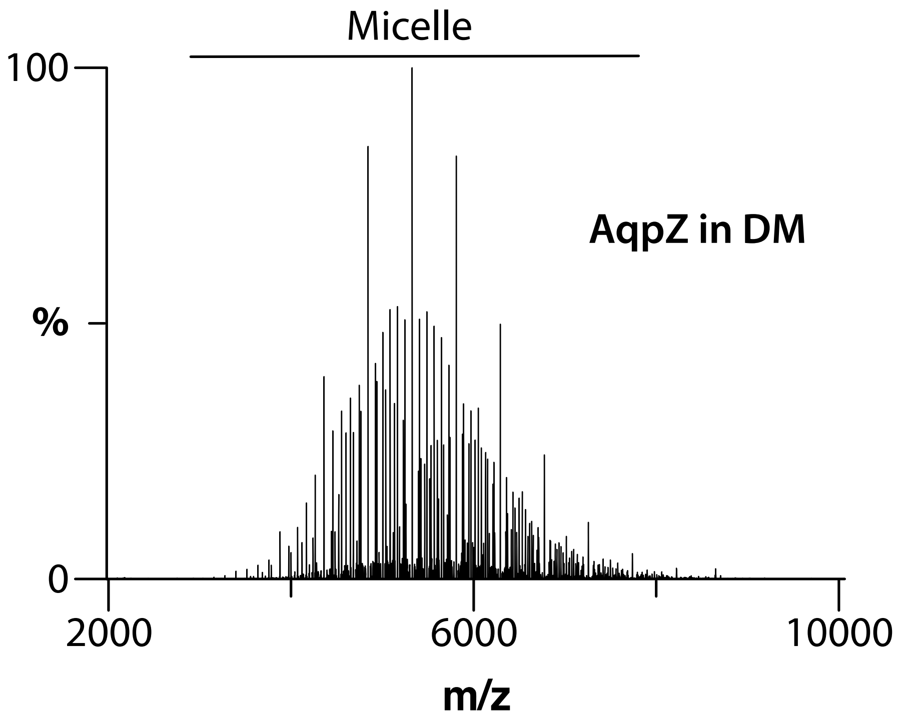

Figure S1. Mass spectrum of AqpZ (1 µM) in DM. The mass spectrum corresponds to DM micelles with no signal for AqpZ.

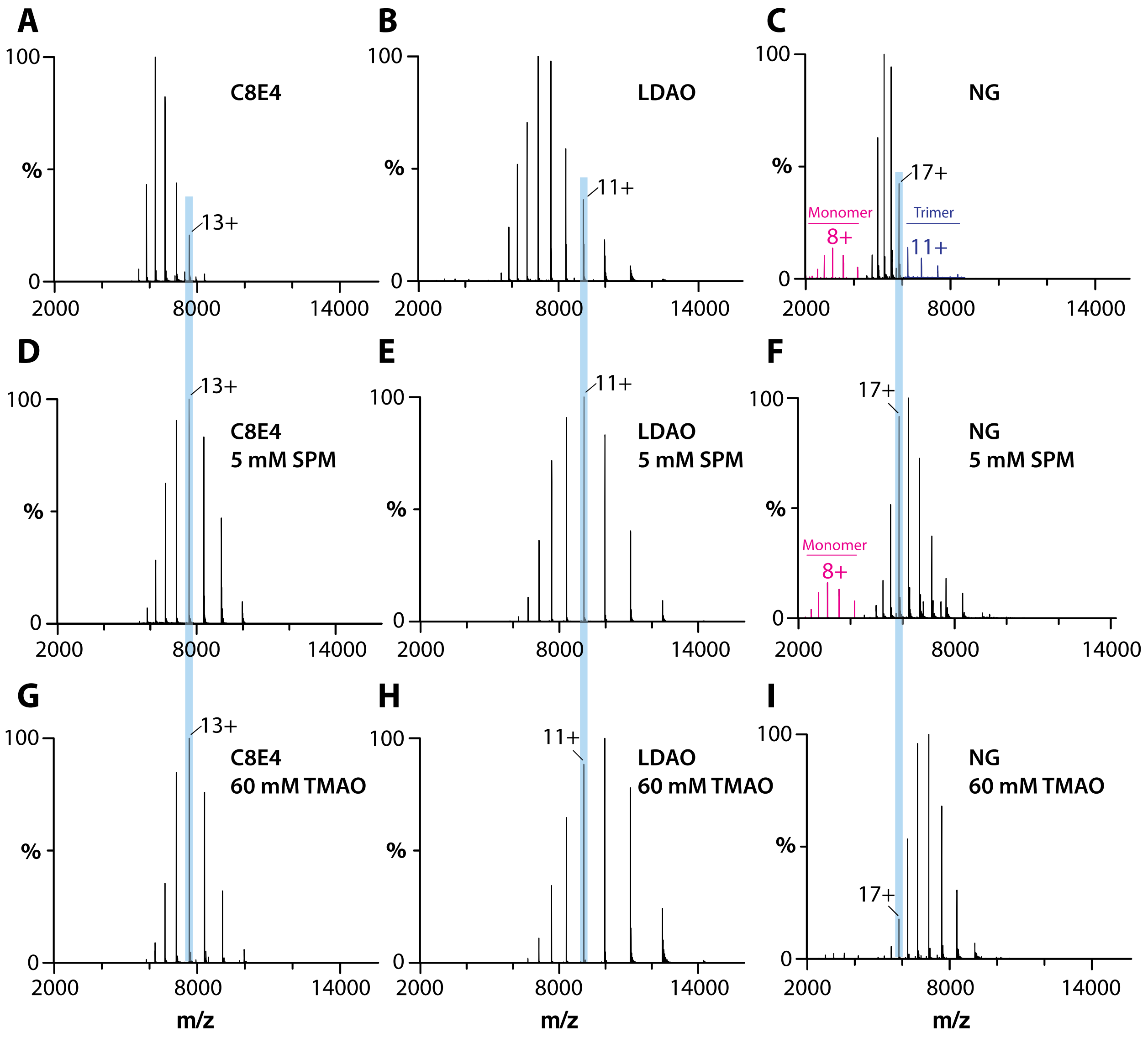

Figure S2. Mass spectra of AqpZ in different detergents with SPM and TMAO. A-C) Mass spectra of 1 μM AqpZ in A) C8E4, B) LDAO, and C) NG without any charge-reducing molecule. D-F) Mass spectra of 1 μM AqpZ in D) C8E4, E) LDAO, and F) NG in the presence of 5 mM SPM. G-I) Mass spectra of 1 μM AqpZ in G) C8E4, H) LDAO, and I) NG in the presence of 60 mM TMAO.

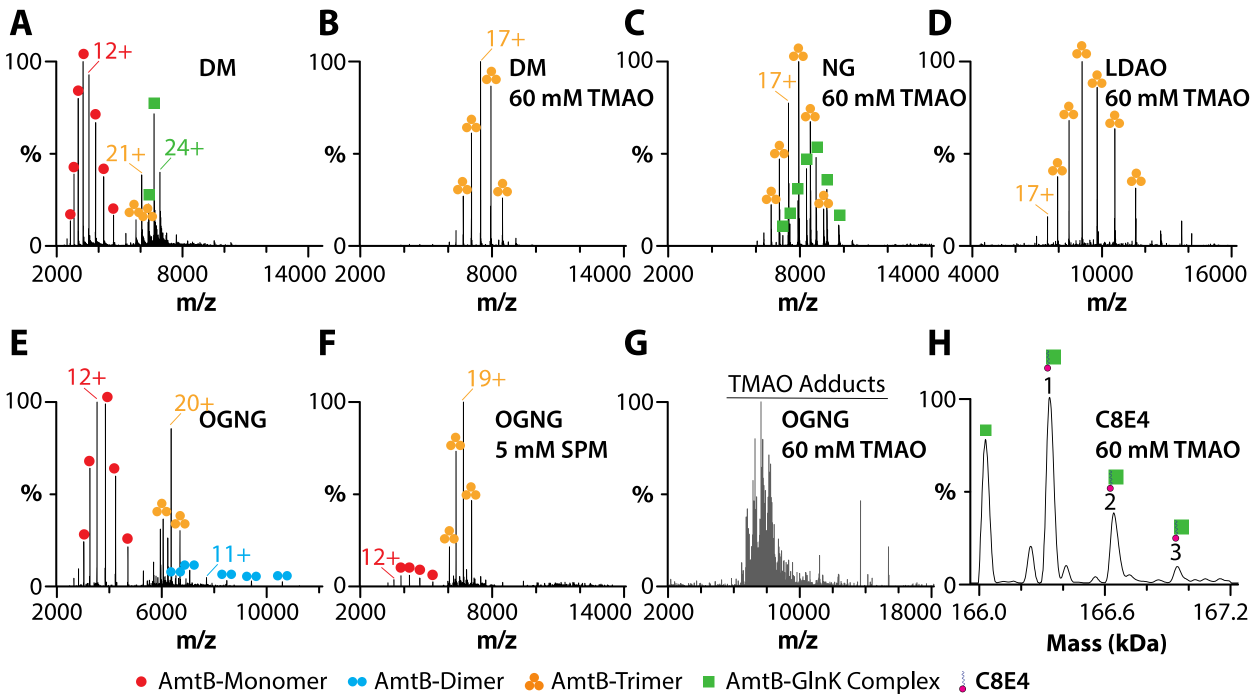

Figure S3. AmtB-GlnK in different detergent environments. Mass spectra of 2 µM AmtB-GlnK in A) DM, B) DM with 60 mM TMAO, C) NG with 60 mM TMAO, D) LDAO with 60 mM TMAO, E) OGNG, F) OGNG with 5 mM SPM, G) OGNG with 60 mM TMAO and H) C8E4 with 60 mM TMAO.

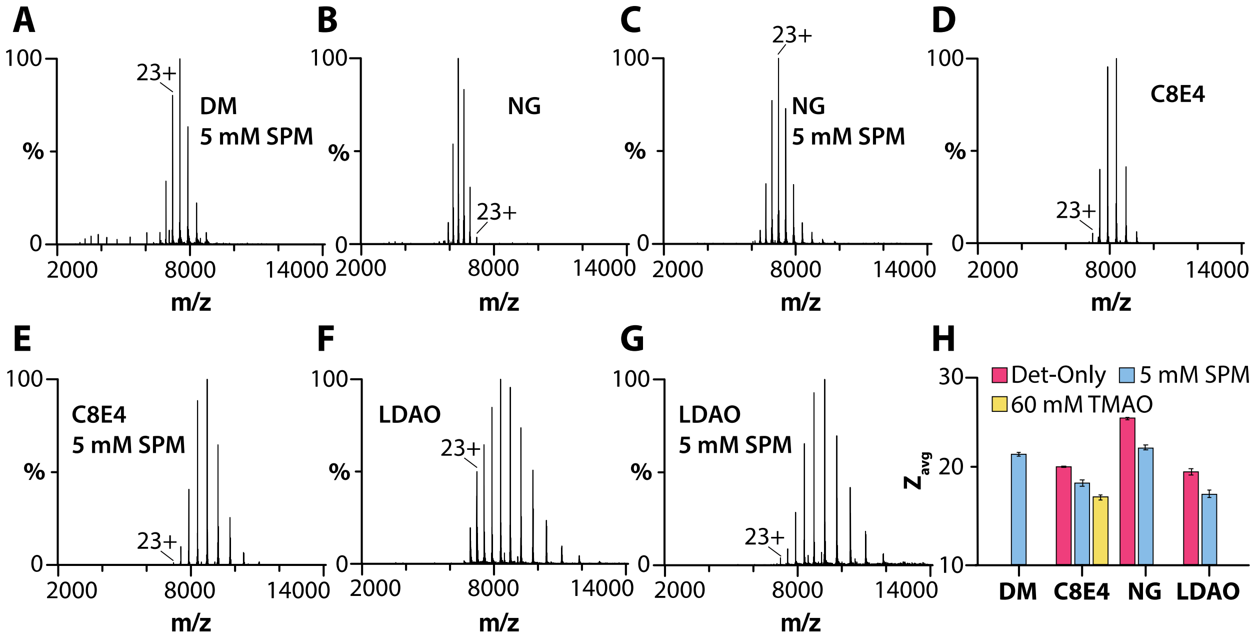

Figure S4. AmtB-GlnK complex in different environments. A-G) Mass spectra of 2 µM AmtB-GlnK in various detergent environments. Shown as described in Figure S1. H) Plot of Z_{avg} for AqpZ in different detergent environments in the presence or absence of charge-reducing molecules. Reported are the mean and standard deviation (*n*=3).

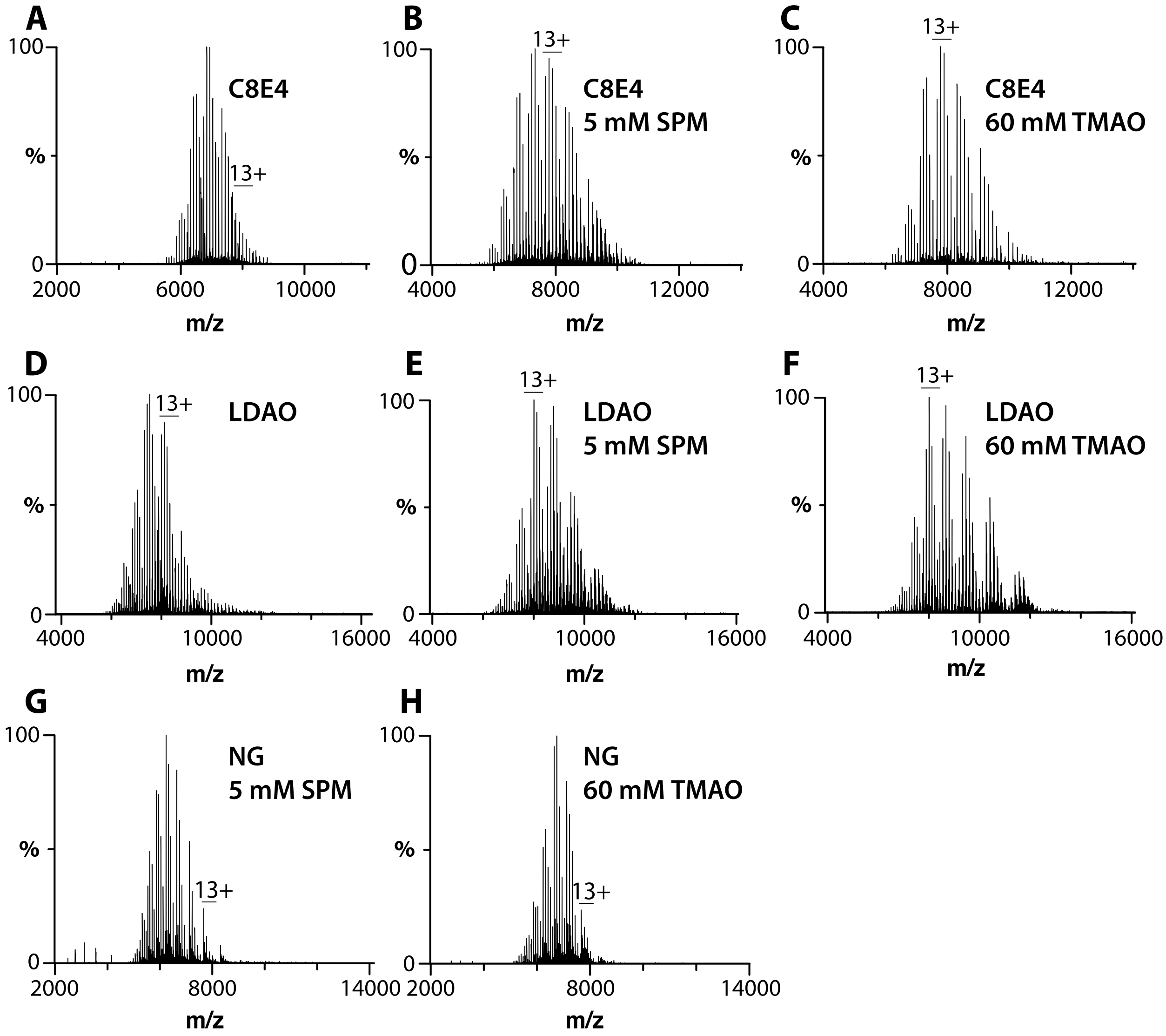

Figure S5. TOCDL binding to AqpZ in different detergents. Mass spectra of 1 µM AqpZ mixed with 25 µM TOCDL in A) C8E4, B) C8E4 with 5 mM SPM, C) C8E4 with 60 mM TMAO, D) LDAO, E) LDAO with 5 mM SPM, F) LDAO with 60 mM TMAO, G) NG with 5 mM SPM and H) NG with 60 mM TMAO.

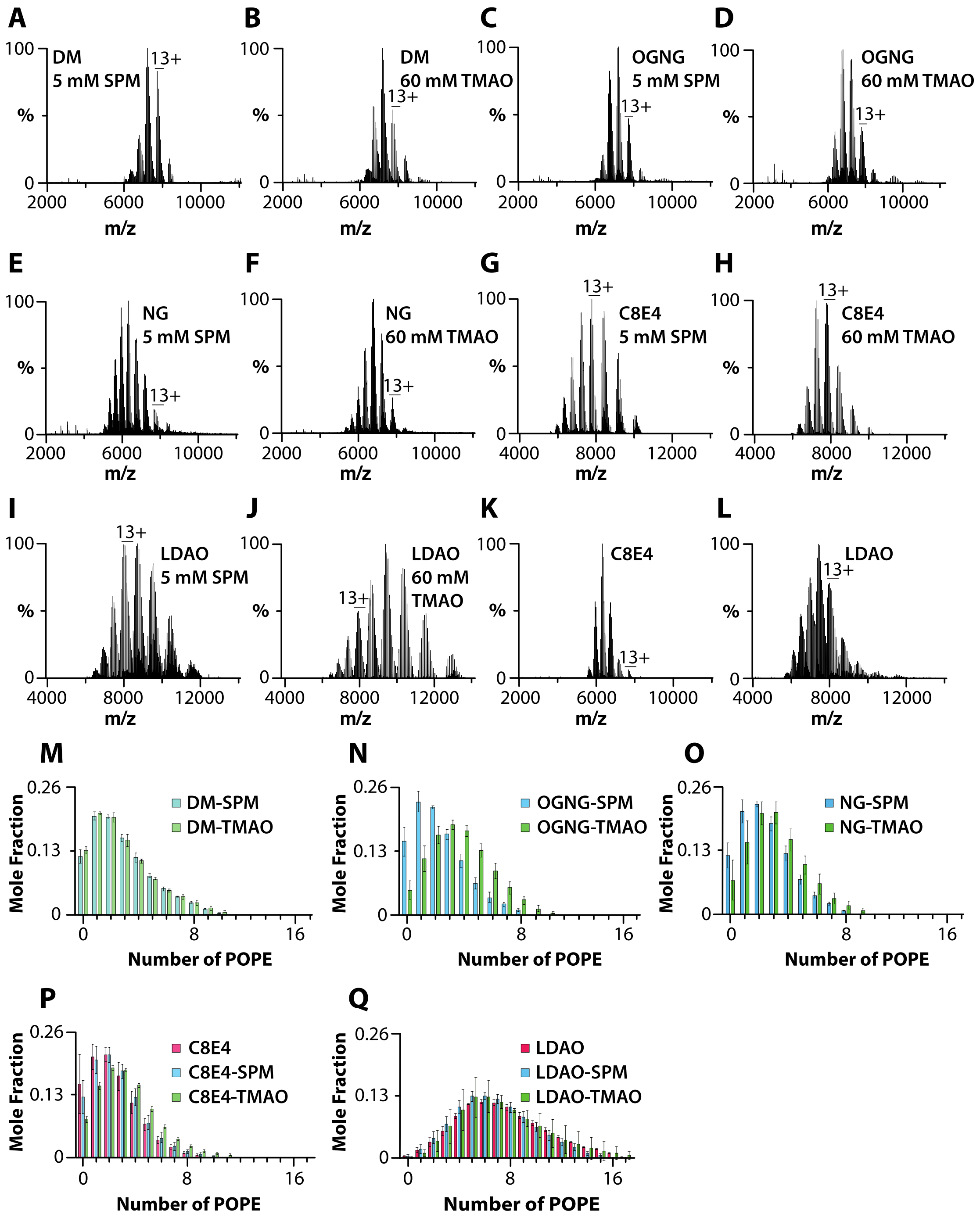

Figure S6. AqpZ-POPE interactions in different detergents. A-L) AqpZ (1 µM) mixed with 50 µM POPE. Shown as described in Figure S5. M-Q) Plot of the mole fraction for different species determined from the deconvolution of the mass spectra shown in A-L. Reported are the mean and standard deviation (*n*=3).

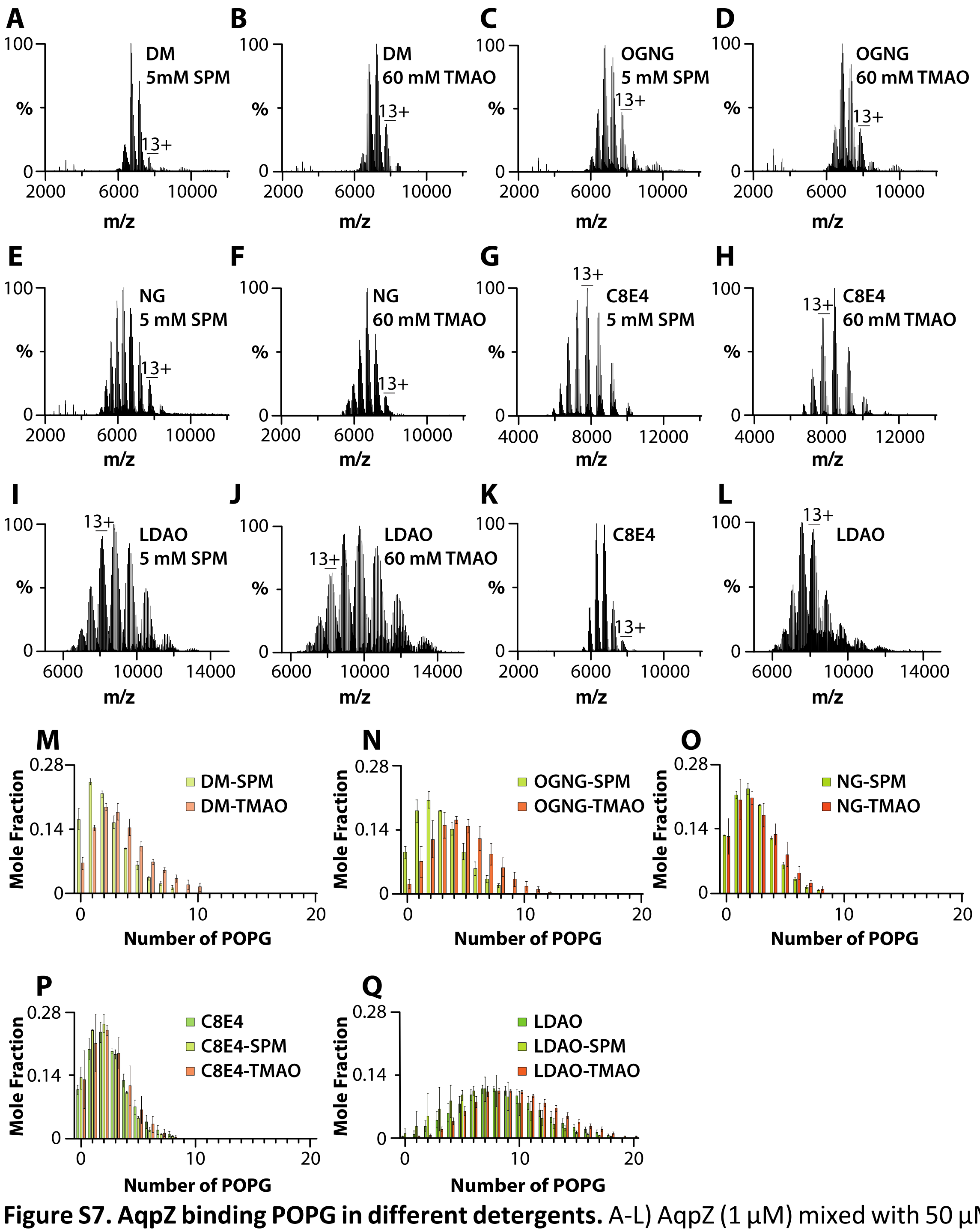

Figure S7. AqpZ binding POPG in different detergents. A-L) AqpZ (1 µM) mixed with 50 µM POPG. Shown as described in Figure S6. M-Q) Plot of the mole fraction for different species determined from the deconvolution of the mass spectra shown in A-L. Reported are the mean and standard deviation (*n*=3).

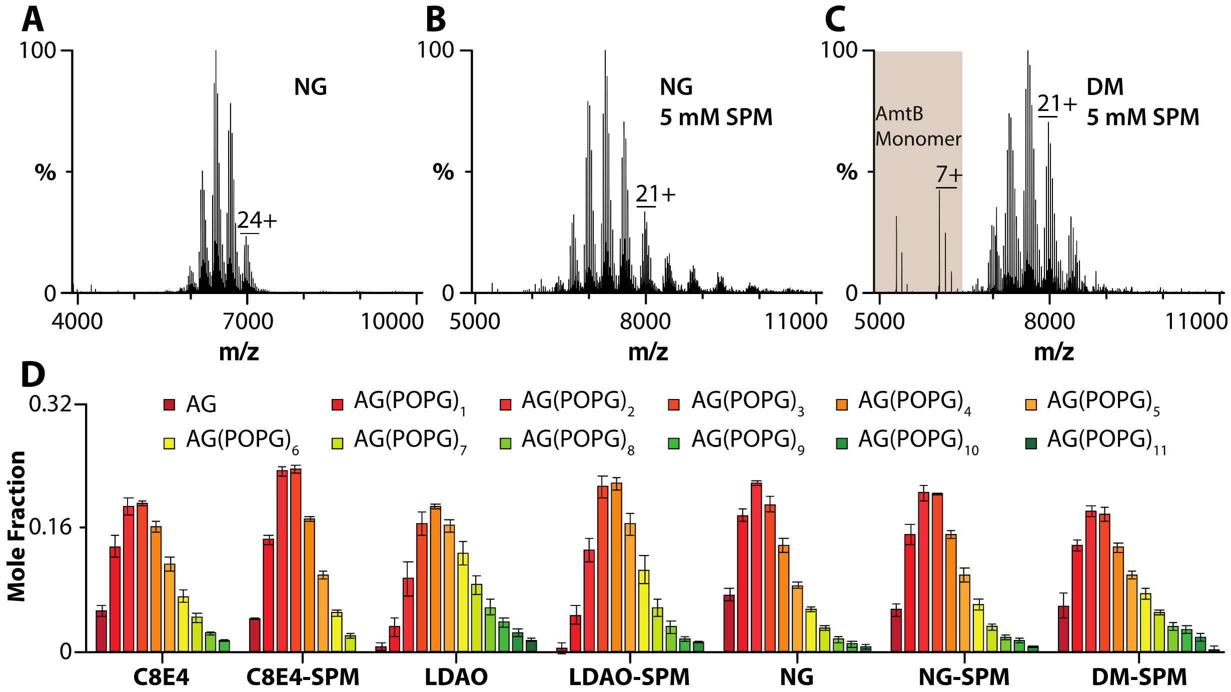

Figure S8. POPG binding to AmtB-GlnK in different detergents. A-C) AmtB-GlnK (2 µM) mixed with 50 µM POPG. Shown as described in Figure 4. D) Plot of the mole fraction for different species determined from the deconvolution of the mass spectra in different environments. Reported are the mean and standard deviation (*n*=3).

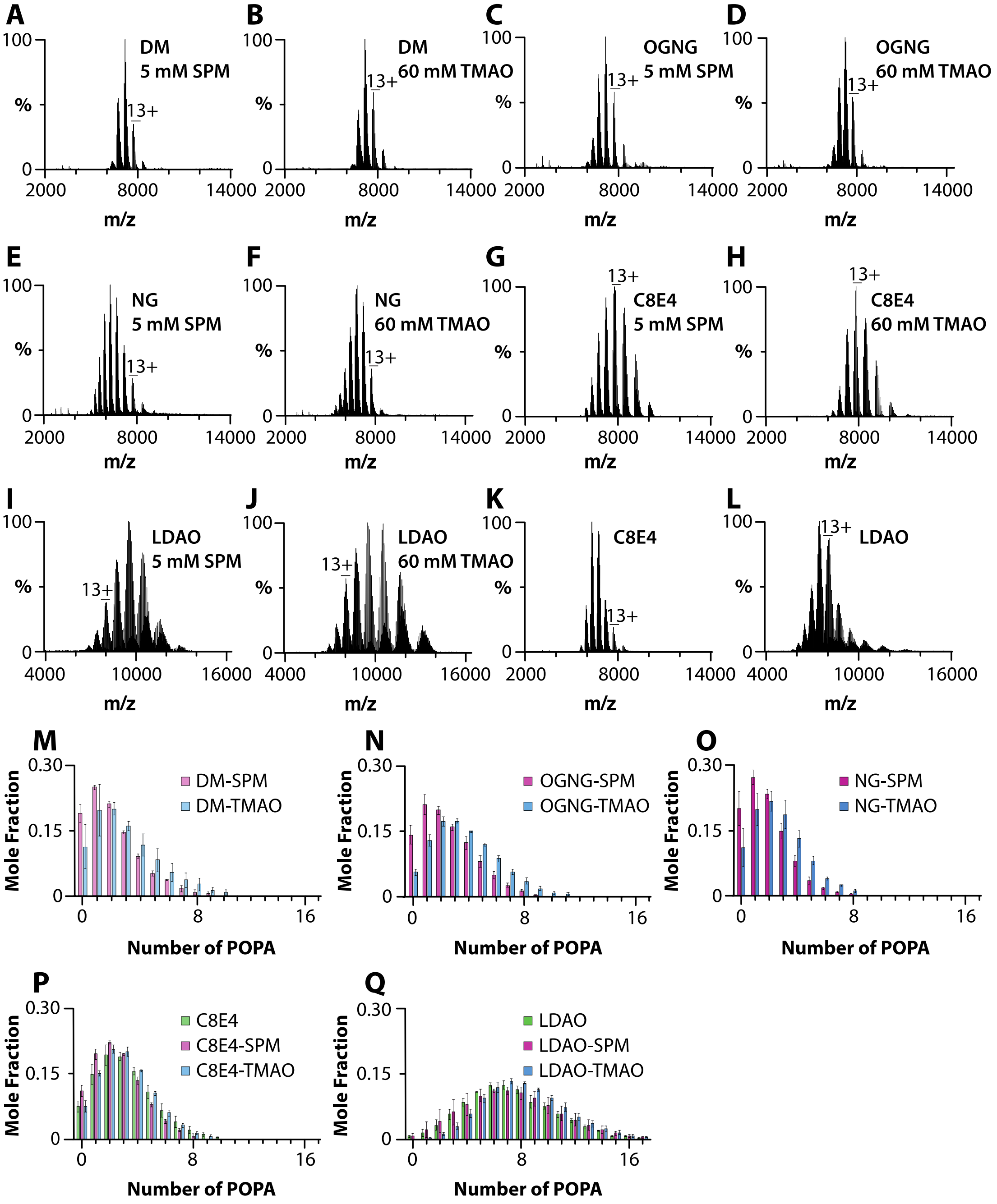

Figure S9. POPA binding to AqpZ in different detergents. A-L) AqpZ (1 µM) mixed with 50 µM POPA. Shown as described in Figure S6. M-Q) Plot of the mole fraction for different species determined from the deconvolution of the mass spectra shown in A-L. Reported are the mean and standard deviation (*n*=3).

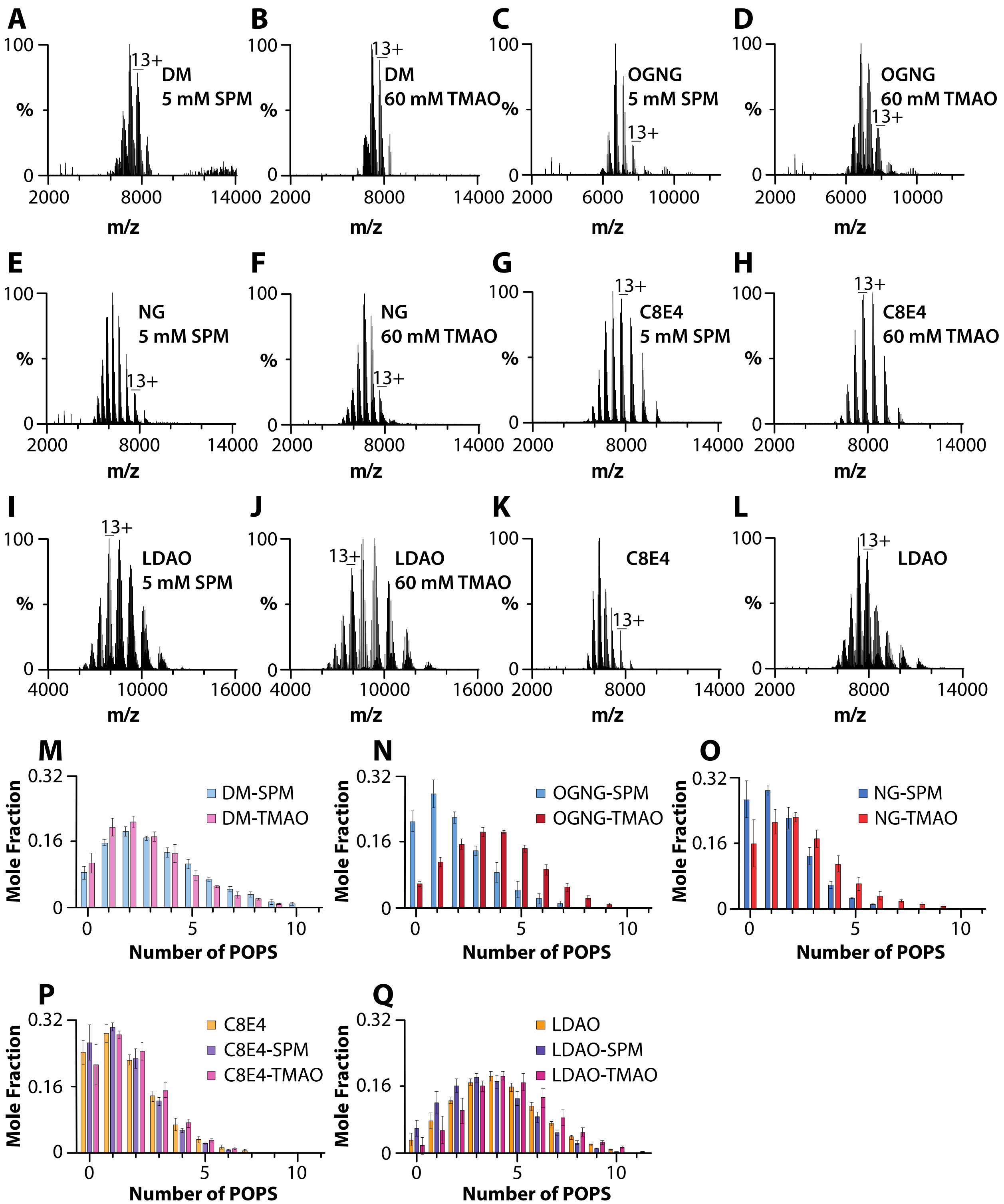

Figure S10. POPS binding to AqpZ in different detergents. A-L) AqpZ (1 µM) was mixed with 50 µM POPS. Shown as described in Figure S6. M-Q) Plot of the mole fraction for different species determined from the deconvolution of the mass spectra shown in A-L. Reported are the mean and standard deviation (*n*=3).

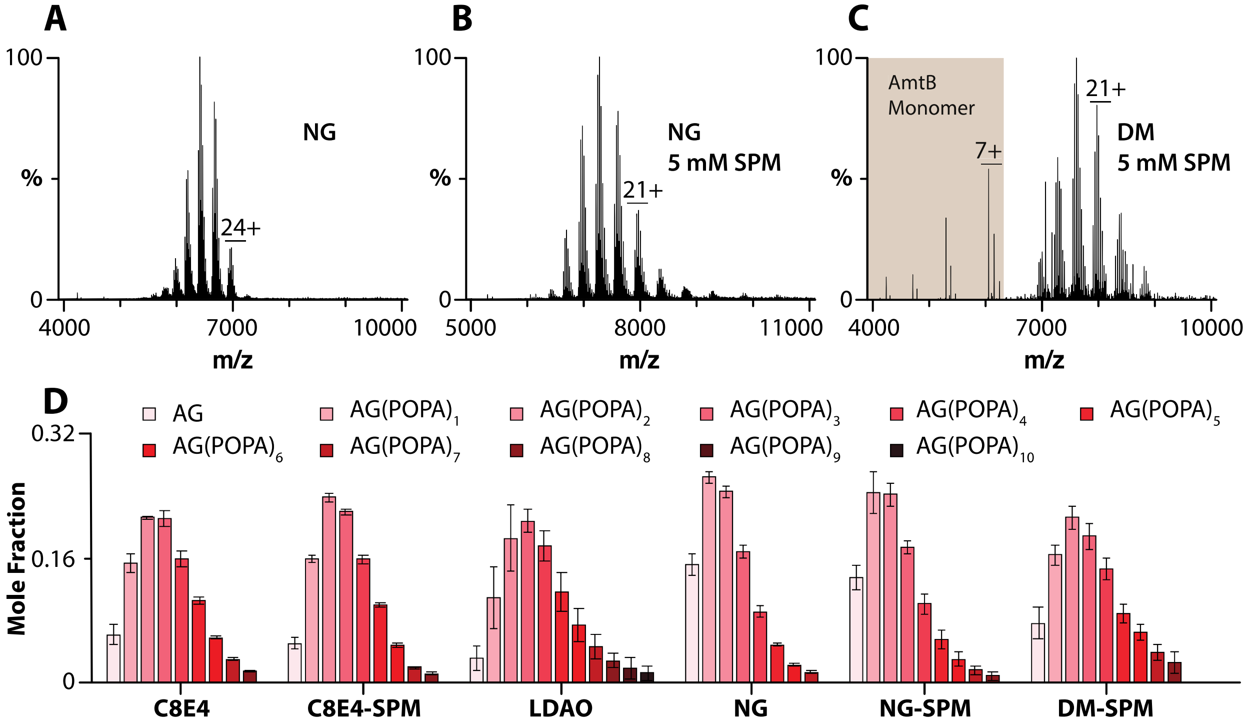

Figure S11. AmtB-GlnK binding POPA in different detergents. A-C) AmtB-GlnK (2 µM) mixed with 50 µM POPA. Shown as described in Figure 4. D) Plot of the mole fraction for different species determined from the deconvolution of the mass spectra shown in different environments. Reported are the mean and standard deviation (*n*=3).

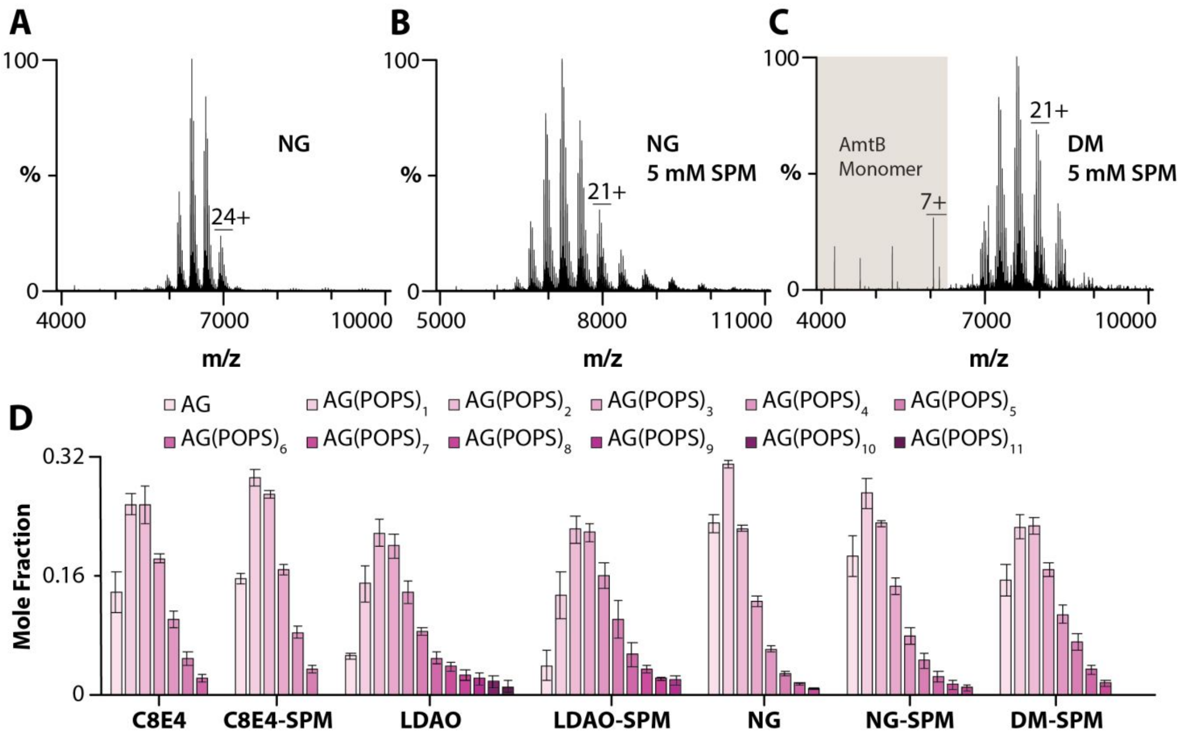

Figure S12. POPS binding to AmtB-GlnK in different detergents. A-C) AmtB-GlnK (2 µM) mixed with 50 µM POPS. Shown as described in Figure 4. D) Plot of the mole fraction for different species determined from the deconvolution of the mass spectra shown in A-C. Reported are the mean and standard deviation (*n*=3).

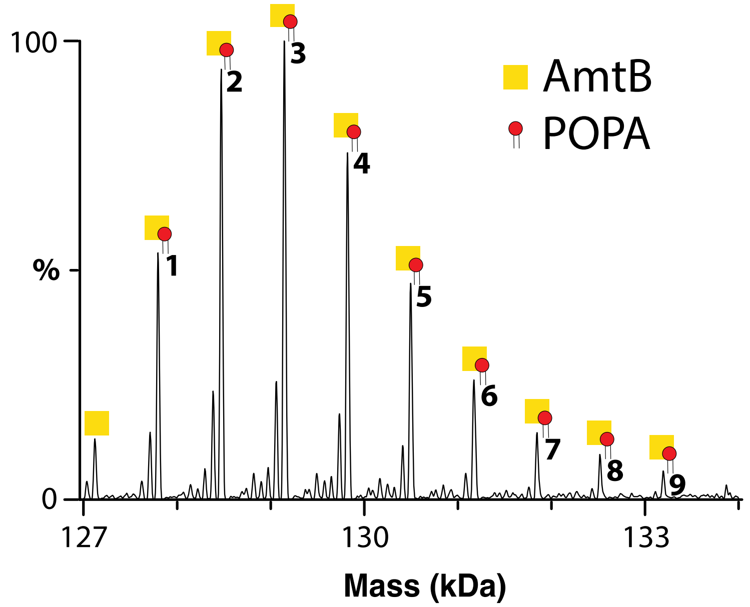

Figure S13. POPA binding to AmtB in LDAO with spermine. Deconvoluted mass spectra of 2 µM AmtB-GlnK in LDAO and 5 mM SPM mixed with 50 µM POPA.

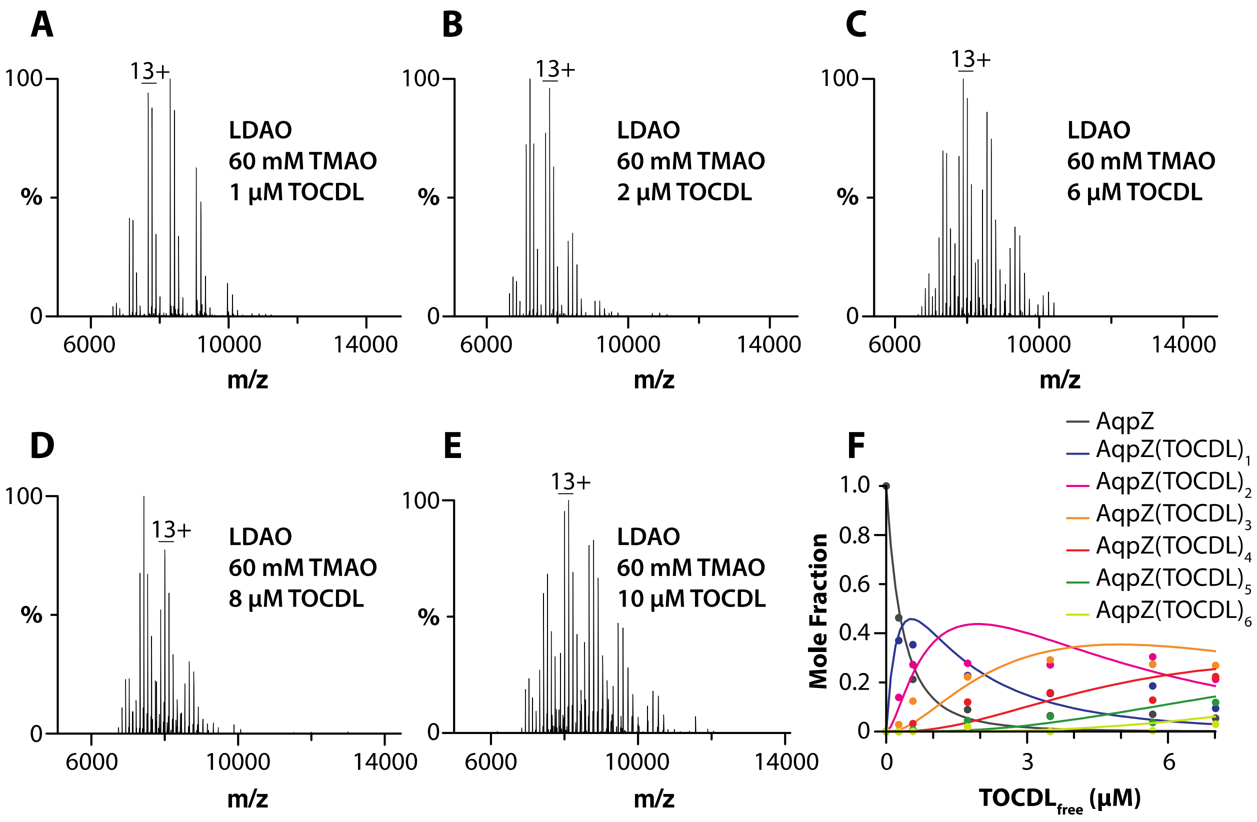

Figure S14. Determination of AqpZ-TOCDL equilibrium binding constants. A-E) AqpZ (1 µM) in LDAO and 60 mM TMAO mixed with different concentrations of TOCDL. F) Plot of mole fraction data (dots) determined from a titration series of TOCDL and subsequent fit of a sequential lipid binding model (lines).

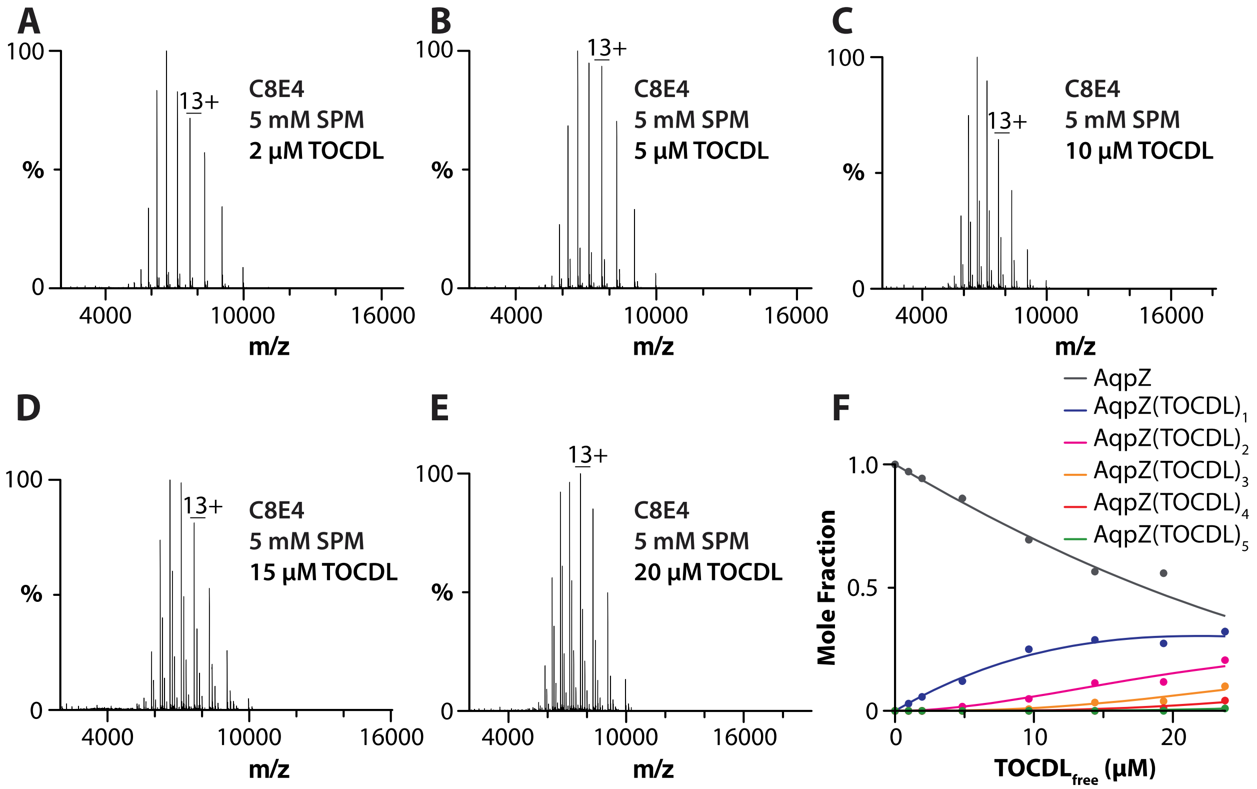

Figure S15. Determination of AqpZ-TOCDL equilibrium binding constants. A-E) AqpZ (1 µM) in C8E4 and 5 mM SPM mixed with different concentrations of TOCDL. F) Plot of mole fraction data (dots) determined from a titration series of TOCDL and subsequent fit of a sequential lipid binding model (lines).

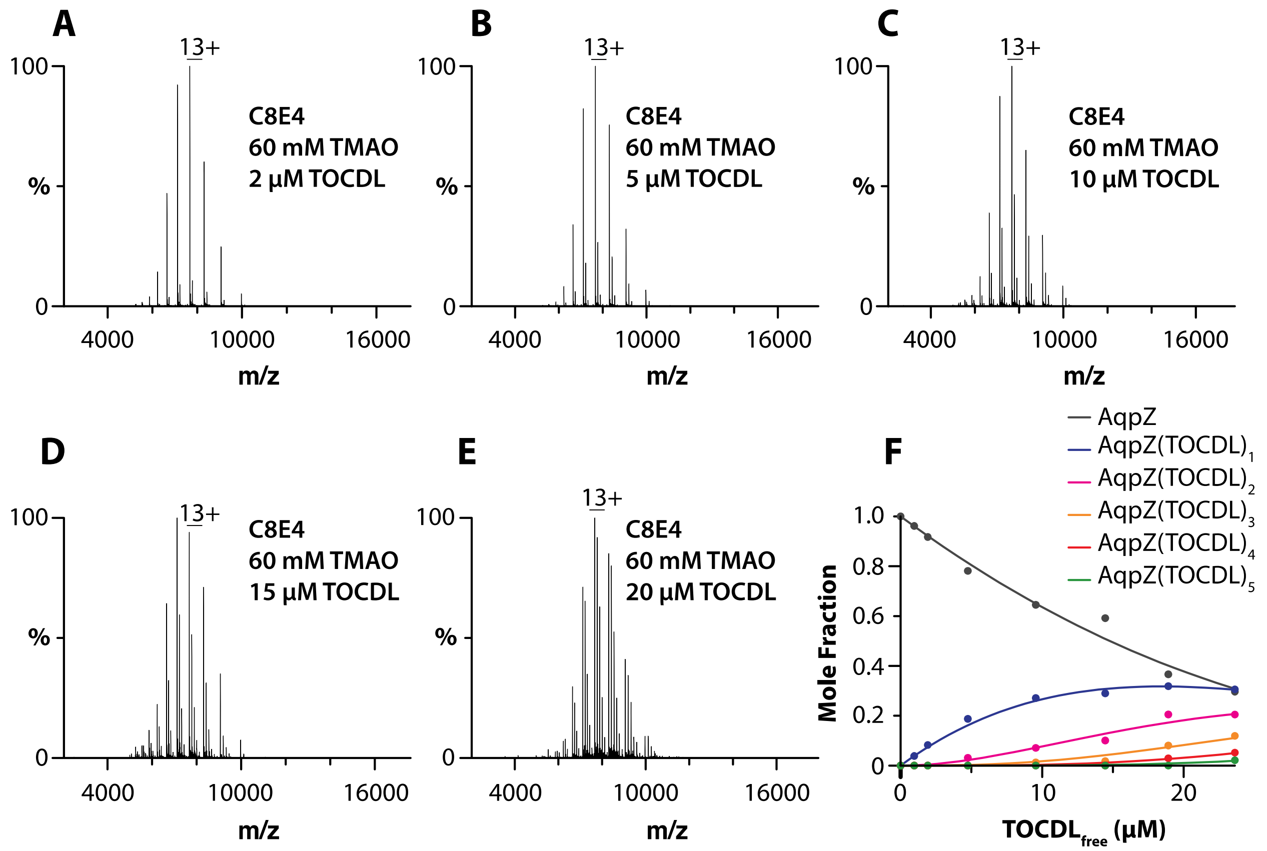

Figure S16. Determination of AqpZ-TOCDL equilibrium binding constants. A-E) AqpZ (1 μM) in C8E4 and 60 mM TMAO mixed with different concentrations of TOCDL. F) Plot of mole fraction data (dots) determined from a titration series of TOCDL and subsequent fit of a sequential lipid binding model (lines).

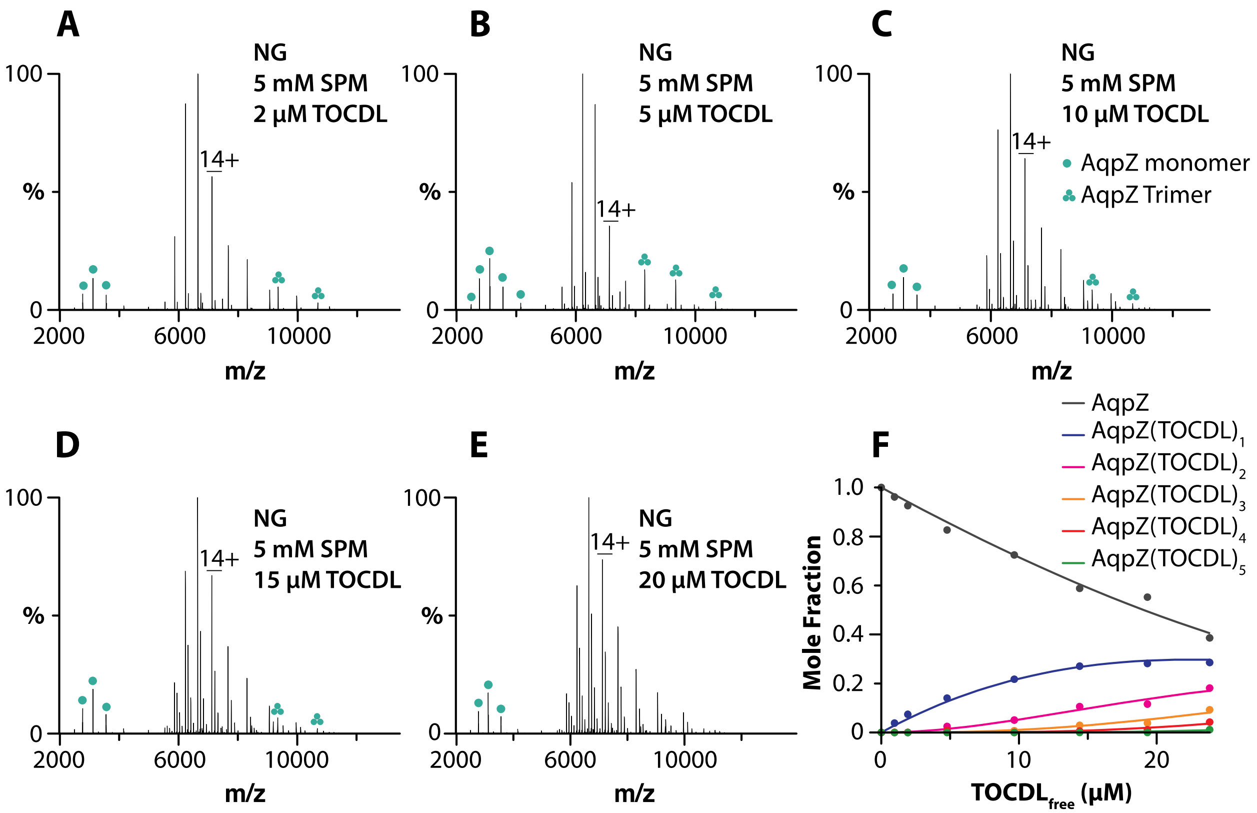

Figure S17. Determination of AqpZ-TOCDL equilibrium binding constants. A-E) AqpZ (1 μM) in NG and 5 mM SPM mixed with different concentrations of TOCDL. F) Plot of mole fraction data (dots) determined from a titration series of TOCDL and subsequent fit of a sequential lipid binding model (lines).

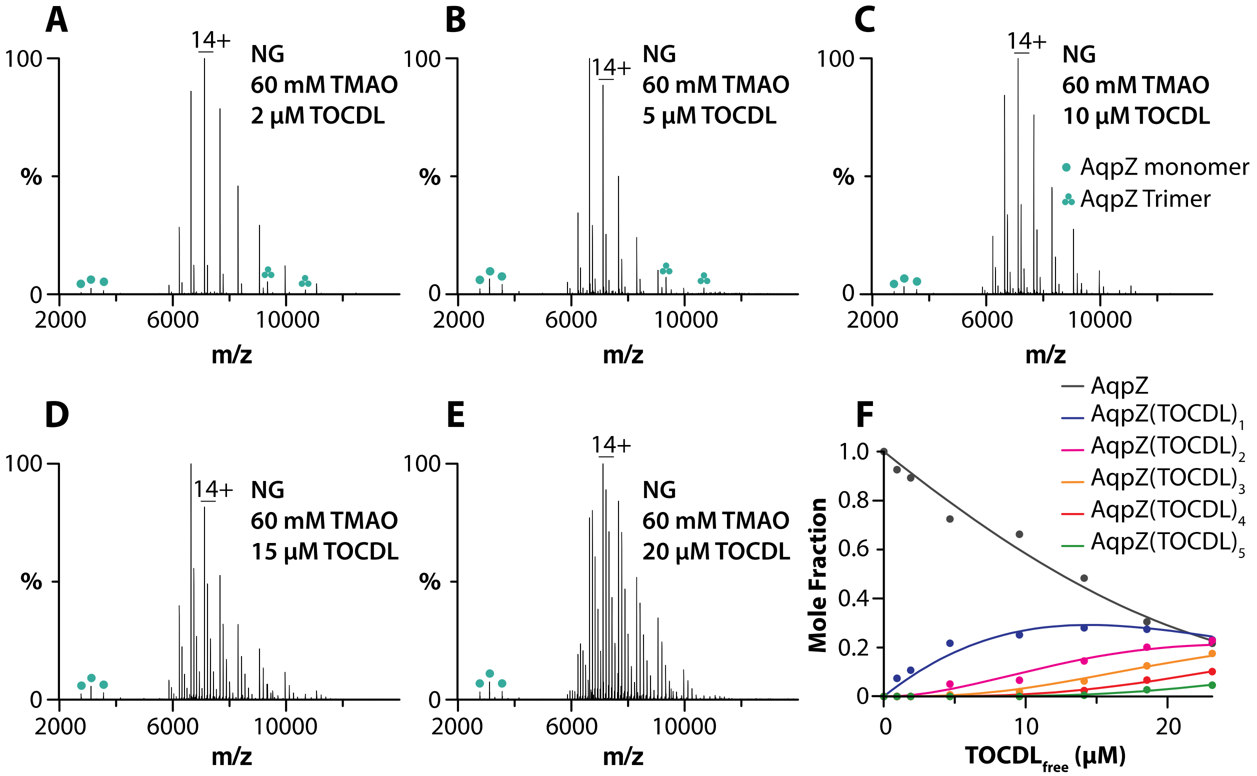

Figure S18. Determination of AqpZ-TOCDL equilibrium binding constants. A-E) AqpZ (1 µM) in NG and 60 mM TMAO mixed with different concentrations of TOCDL. F) Plot of mole fraction data (dots) determined from a titration series of TOCDL and subsequent fit of a sequential lipid binding model (lines).

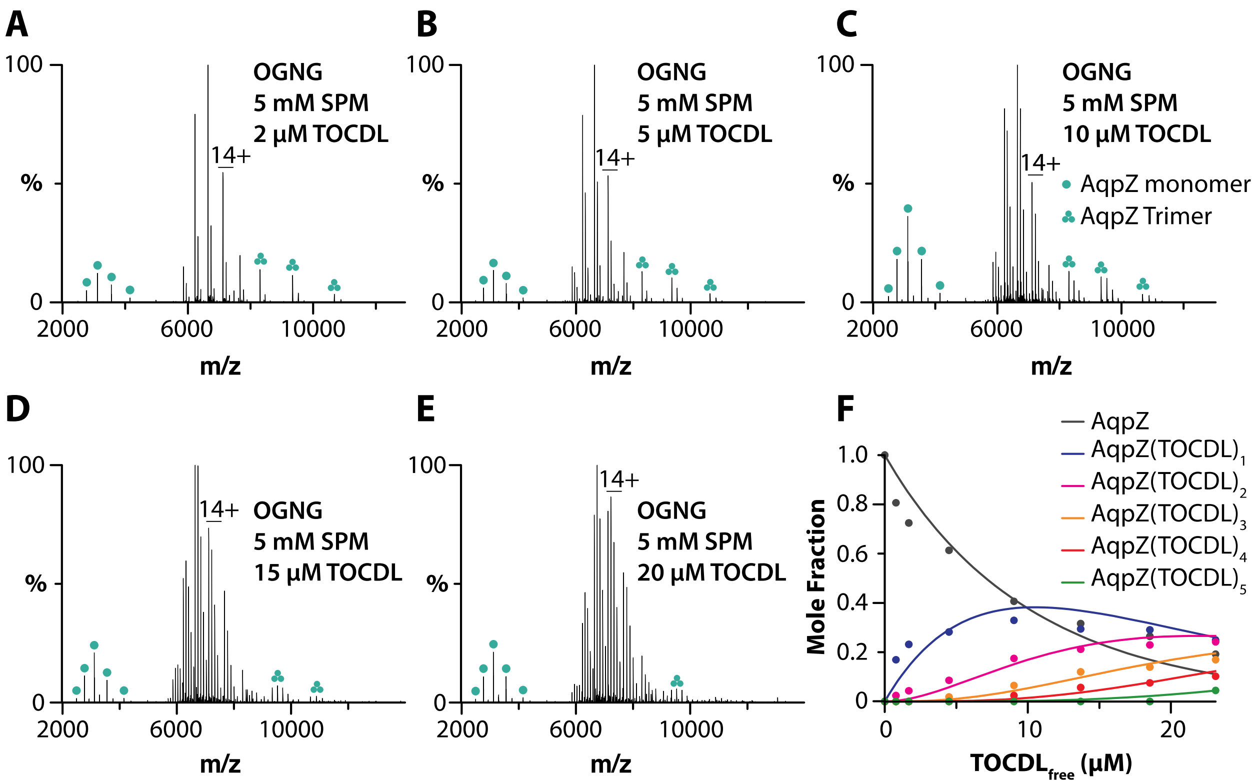

Figure S19. Determination of AqpZ-TOCDL equilibrium binding constants. A-E) AqpZ (1 µM) in OGNG and 5 mM SPM mixed with different concentrations of TOCDL. F) Plot of mole fraction data (dots) determined from a titration series of TOCDL and subsequent fit of a sequential lipid binding model (lines).

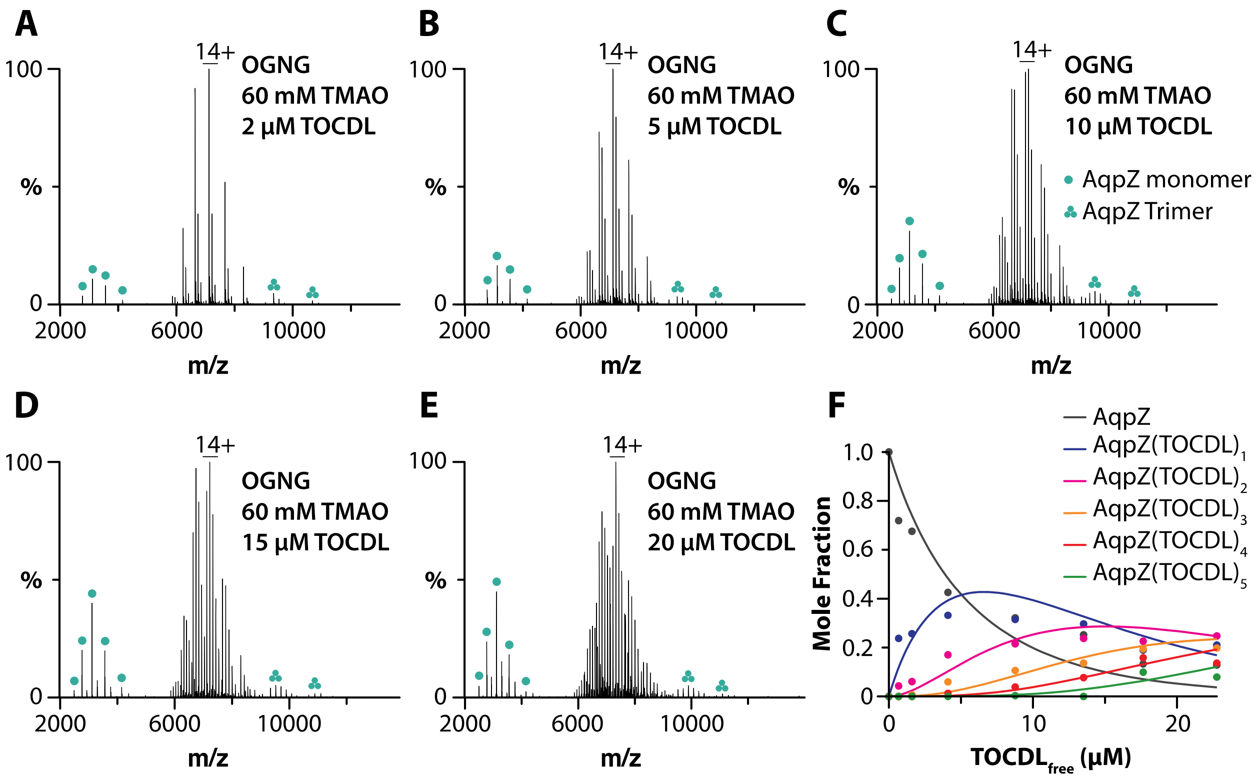

Figure S20. Determination of AqpZ-TOCDL equilibrium binding constants. A-E) AqpZ (1 μM) in OGNG and 60 mM TMAO mixed with different concentrations of TOCDL. F) Plot of mole fraction data (dots) determined from a titration series of TOCDL and subsequent fit of a sequential lipid binding model (lines).

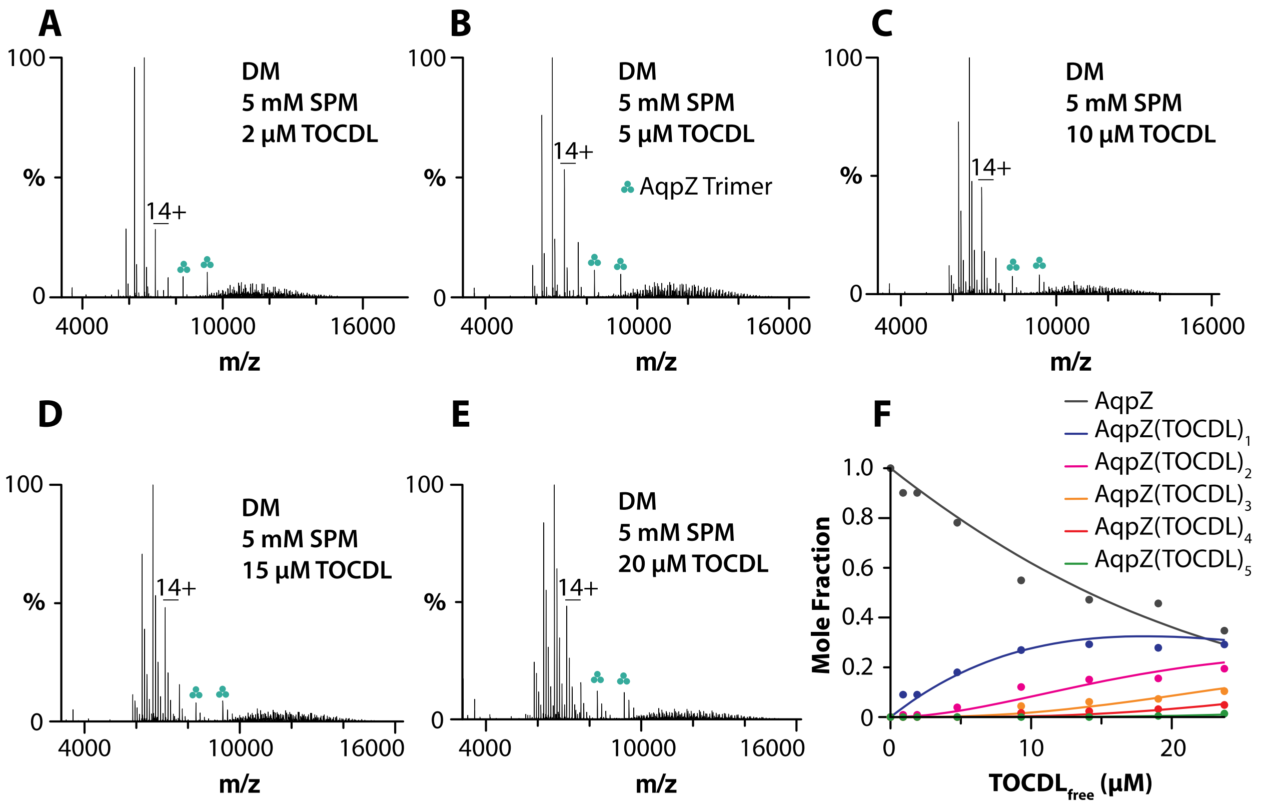

Figure S21. Determination of AqpZ-TOCDL equilibrium binding constants. A-E) AqpZ (1 µM) in DM and 5 mM SPM mixed with different concentrations of TOCDL. F) Plot of mole fraction data (dots) determined from a titration series of TOCDL and subsequent fit of a sequential lipid binding model (lines).

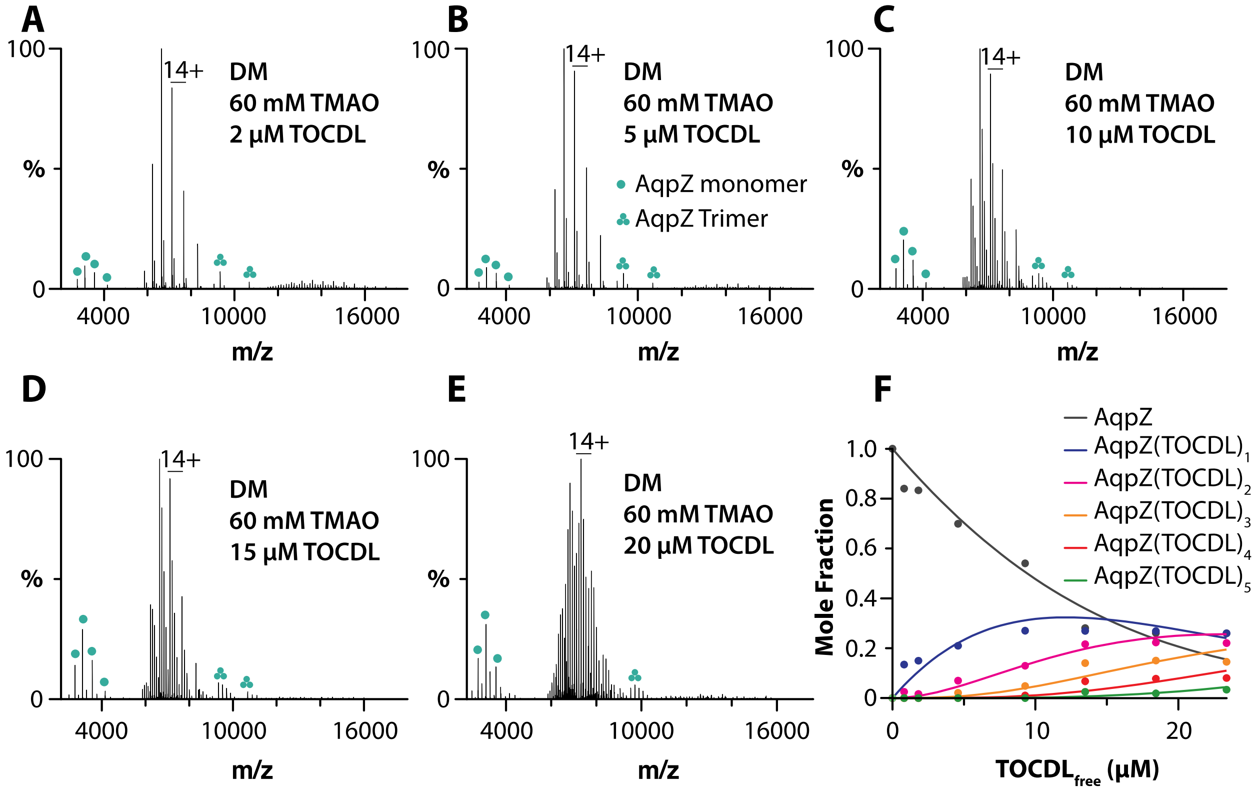

Figure S22. Determination of AqpZ-TOCDL equilibrium binding constants. A-E) AqpZ (1 μM) in DM and 60 mM TMAO mixed with different concentrations of TOCDL. F) Plot of mole fraction data (dots) determined from a titration series of TOCDL and subsequent fit of a sequential lipid binding model (lines).

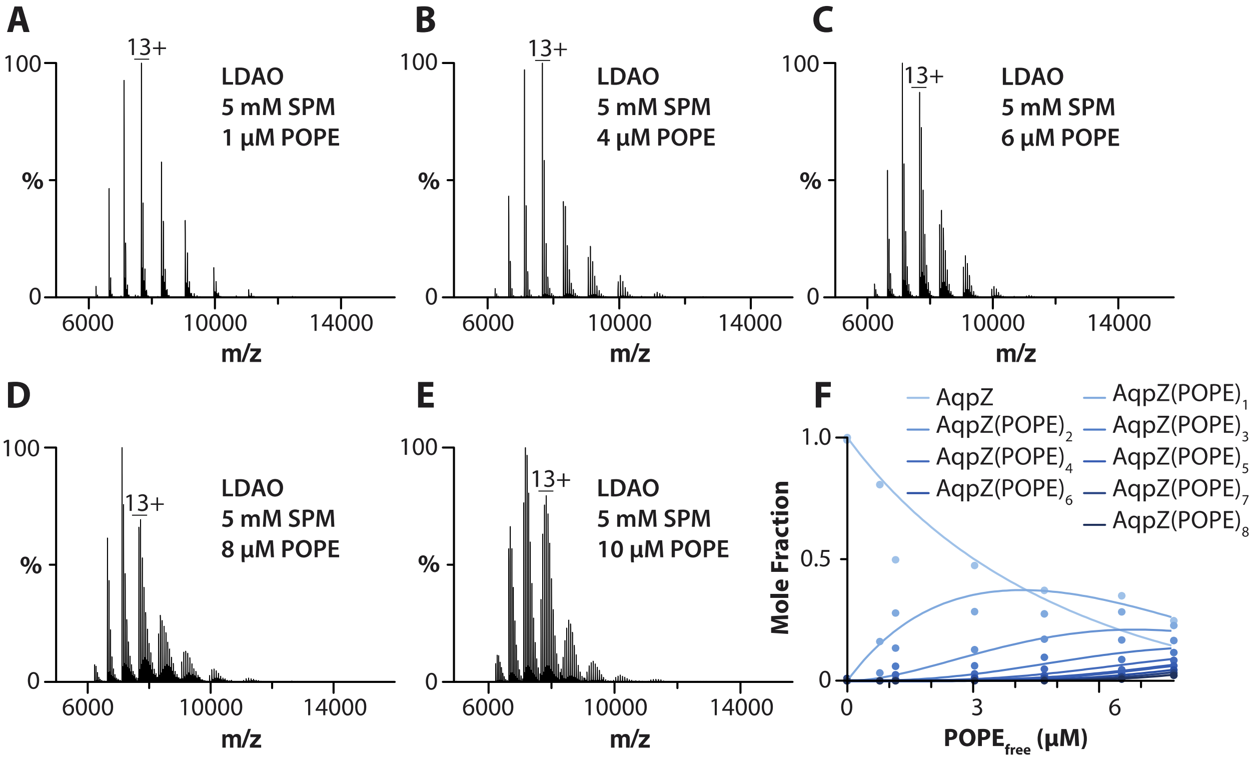

Figure S23. Determination of AqpZ-POPE equilibrium binding constants. A-E) AqpZ (1 µM) in LDAO and 5 mM SPM mixed with different concentrations of POPE. F) Plot of mole fraction data (dots) determined from a titration series of POPE and subsequent fit of a sequential lipid binding model (lines).

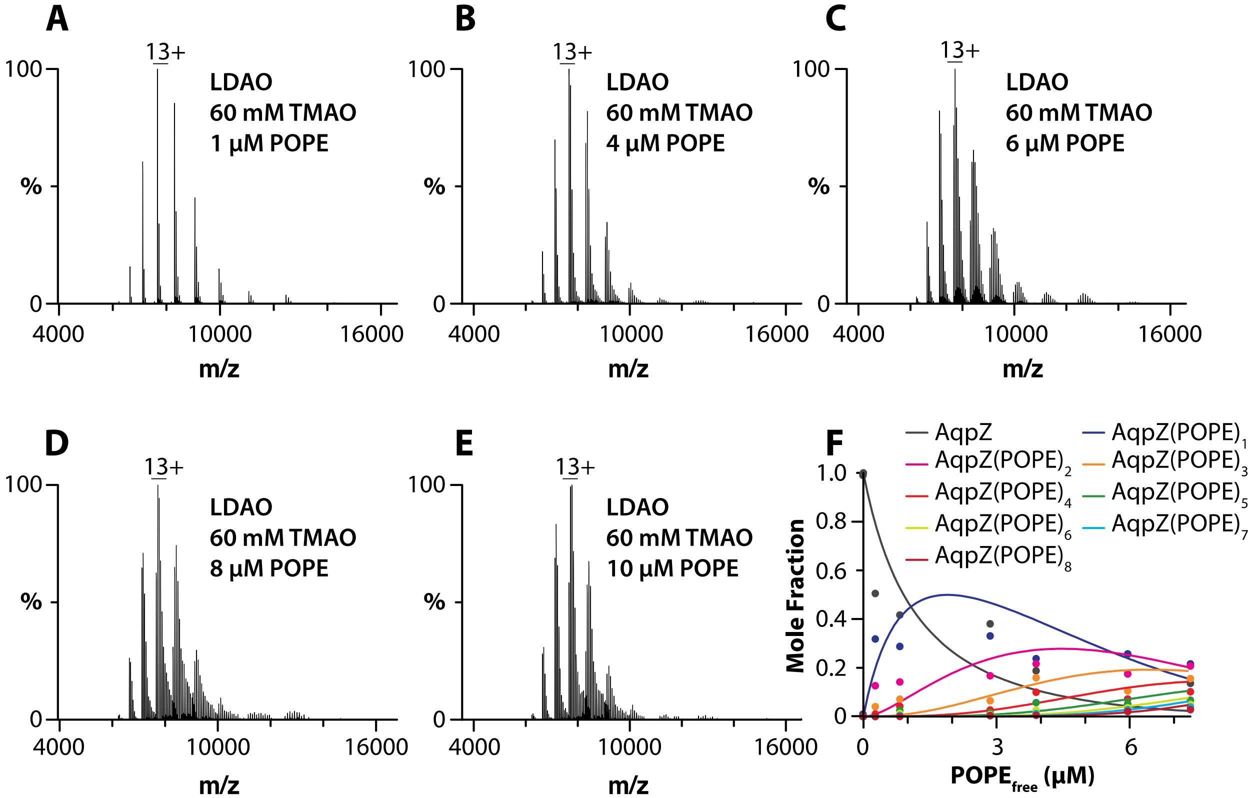

Figure S24. Determination of AqpZ-POPE equilibrium binding constants. A-E) AqpZ (1 μM) in LDAO and 60 mM TMAO mixed with different concentrations of POPE. F) Plot of mole fraction data (dots) determined from a titration series of POPE and subsequent fit of a sequential lipid binding model (lines).

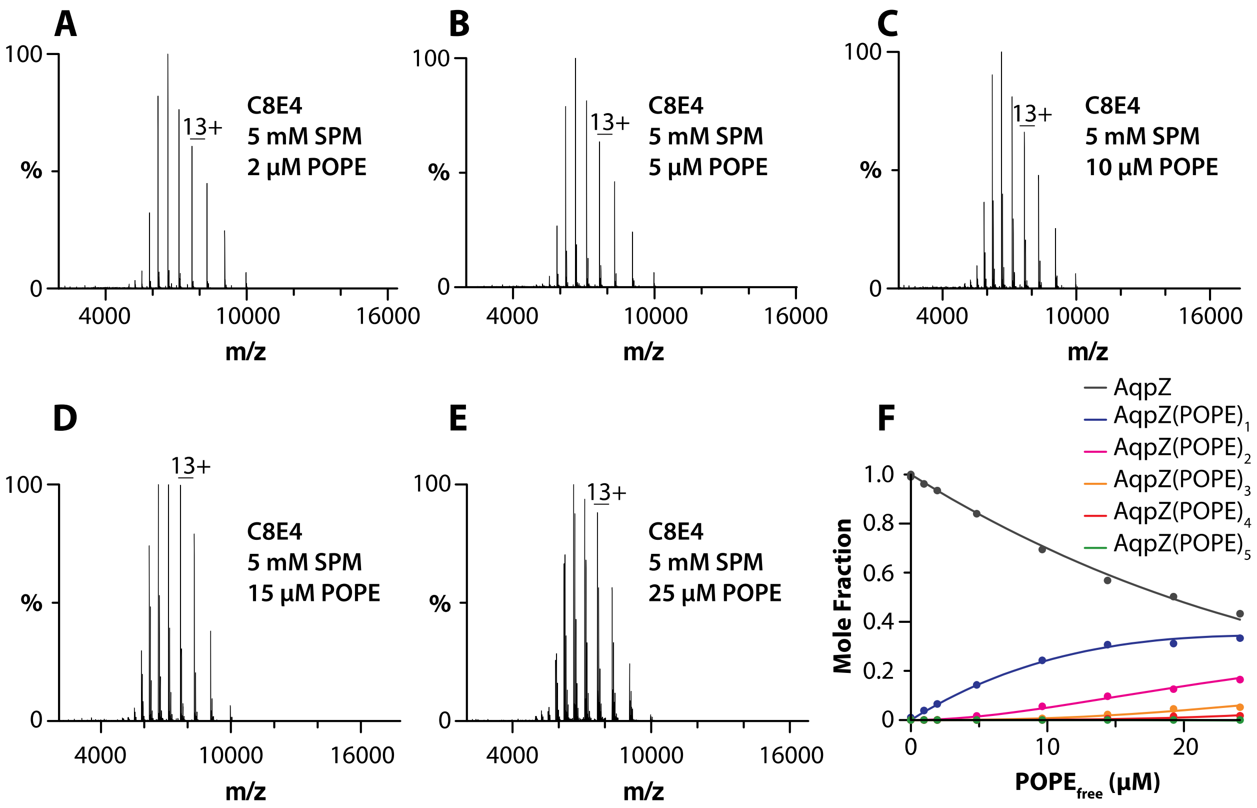

Figure S25. Determination of AqpZ-POPE equilibrium binding constants. A-E) AqpZ (1 µM) in C8E4 and 5 mM SPM mixed with different concentrations of POPE. F) Plot of mole fraction data (dots) determined from a titration series of POPE and subsequent fit of a sequential lipid binding model (lines).

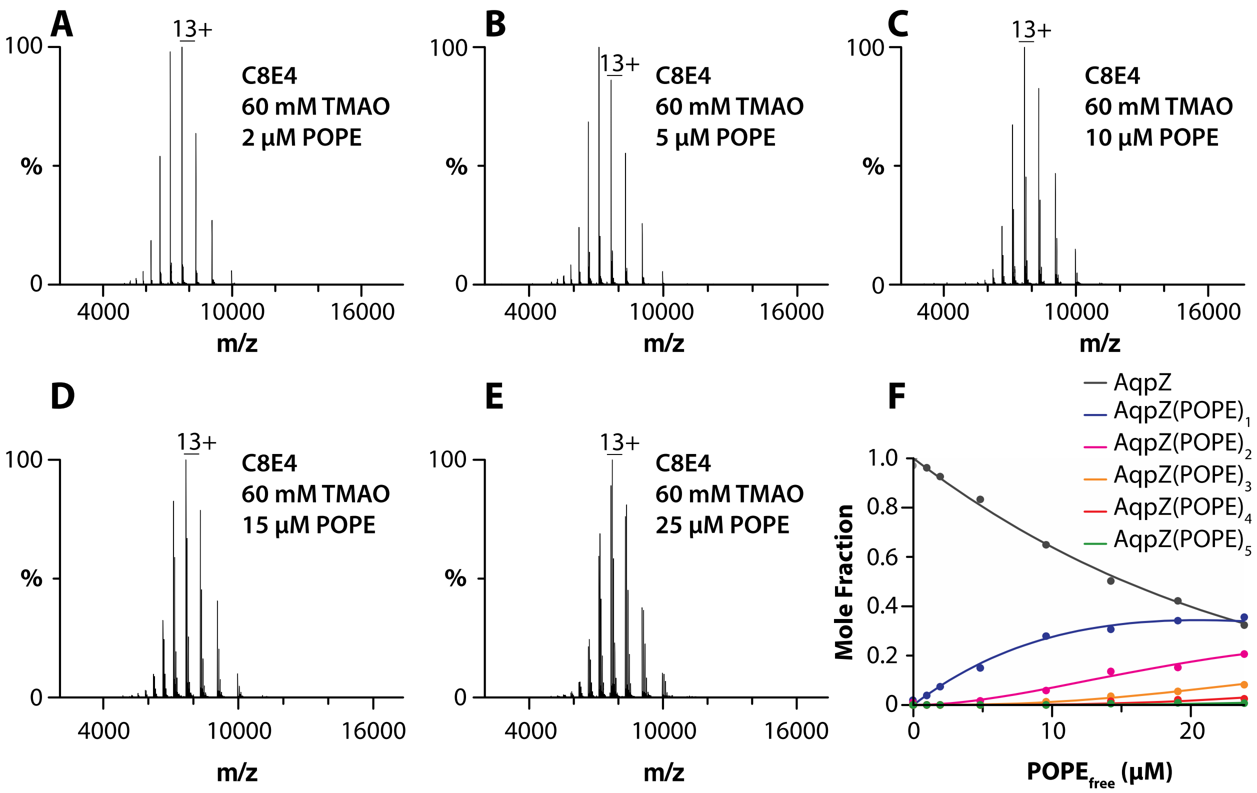

Figure S26. Determination of AqpZ-POPE equilibrium binding constants. A-E) AqpZ (1 µM) in C8E4 and 60 mM TMAO mixed with different concentrations of POPE. F) Plot of mole fraction data (dots) determined from a titration series of POPE and subsequent fit of a sequential lipid binding model (lines).

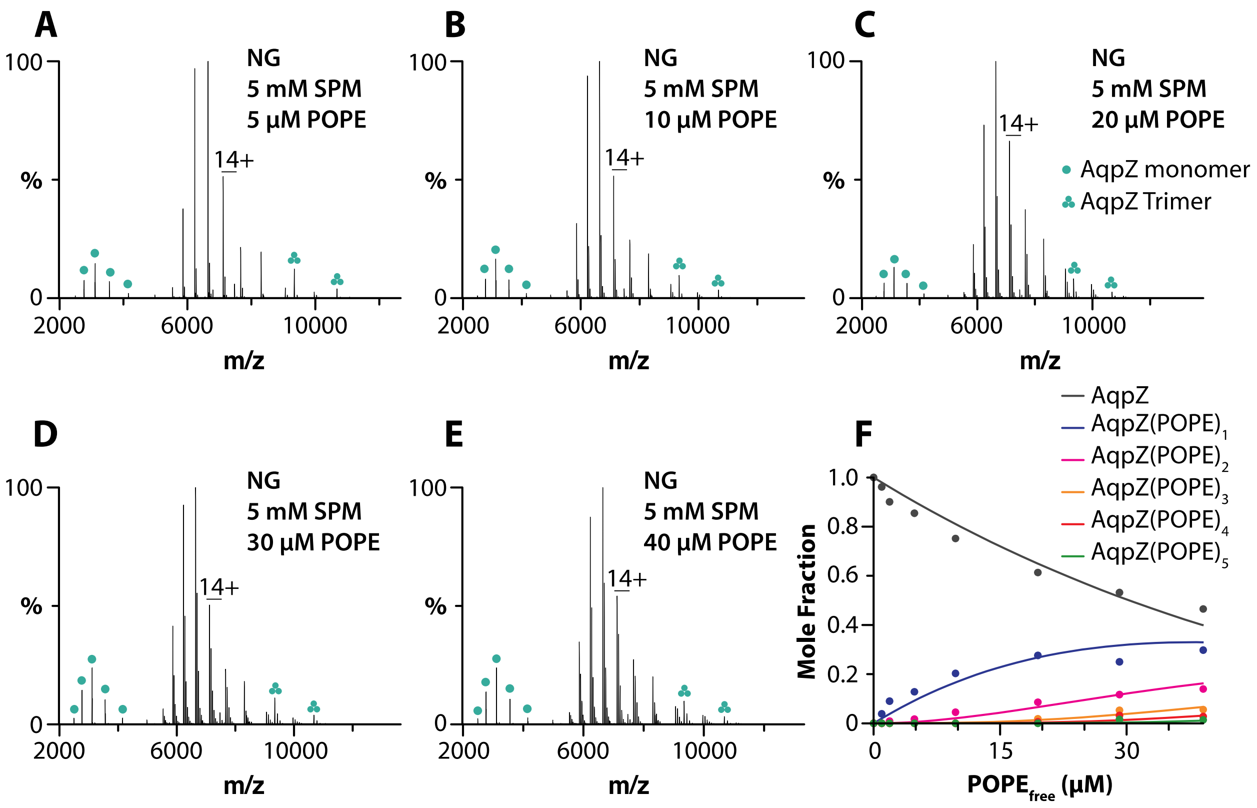

Figure S27. Determination of AqpZ-POPE equilibrium binding constants. A-E) AqpZ (1 µM) in NG and 5 mM SPM mixed with different concentrations of POPE. F) Plot of mole fraction data (dots) determined from a titration series of POPE and subsequent fit of a sequential lipid binding model (lines).

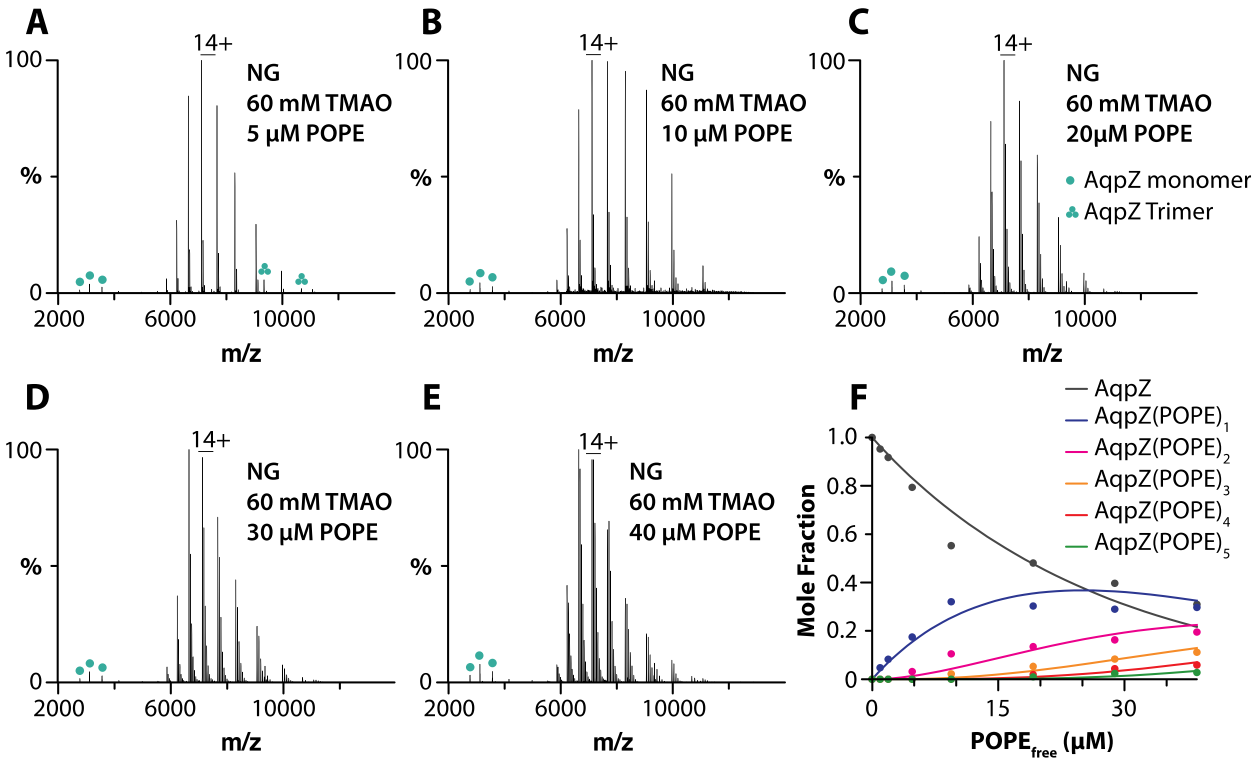

Figure S28. Determination of AqpZ-POPE equilibrium binding constants. A-E) AqpZ (1 µM) in NG and 60 mM TMAO mixed with different concentrations of POPE. F) Plot of mole fraction data (dots) determined from a titration series of POPE and subsequent fit of a sequential lipid binding model (lines).

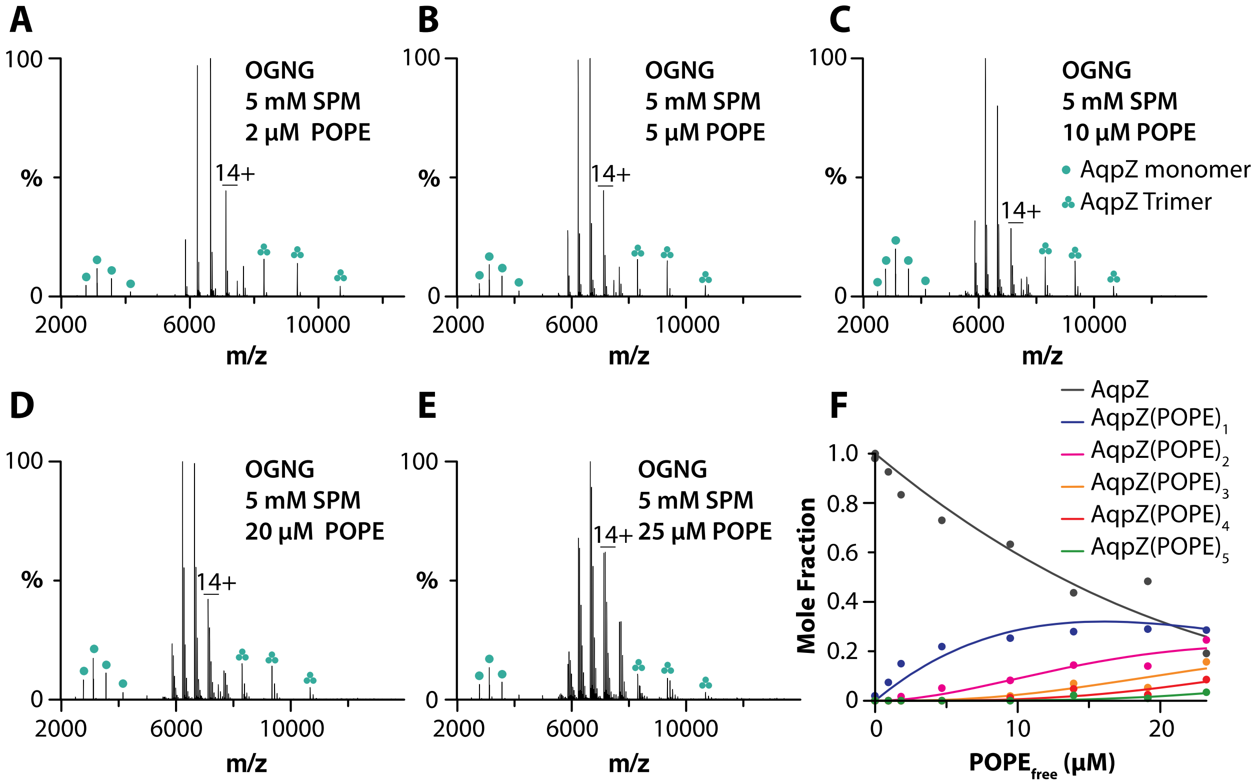

Figure S29. Determination of AqpZ-POPE equilibrium binding constants. A-E) AqpZ (1 µM) in OGNG and 5 mM SPM mixed with different concentrations of POPE. F) Plot of mole fraction data (dots) determined from a titration series of POPE and subsequent fit of a sequential lipid binding model (lines).

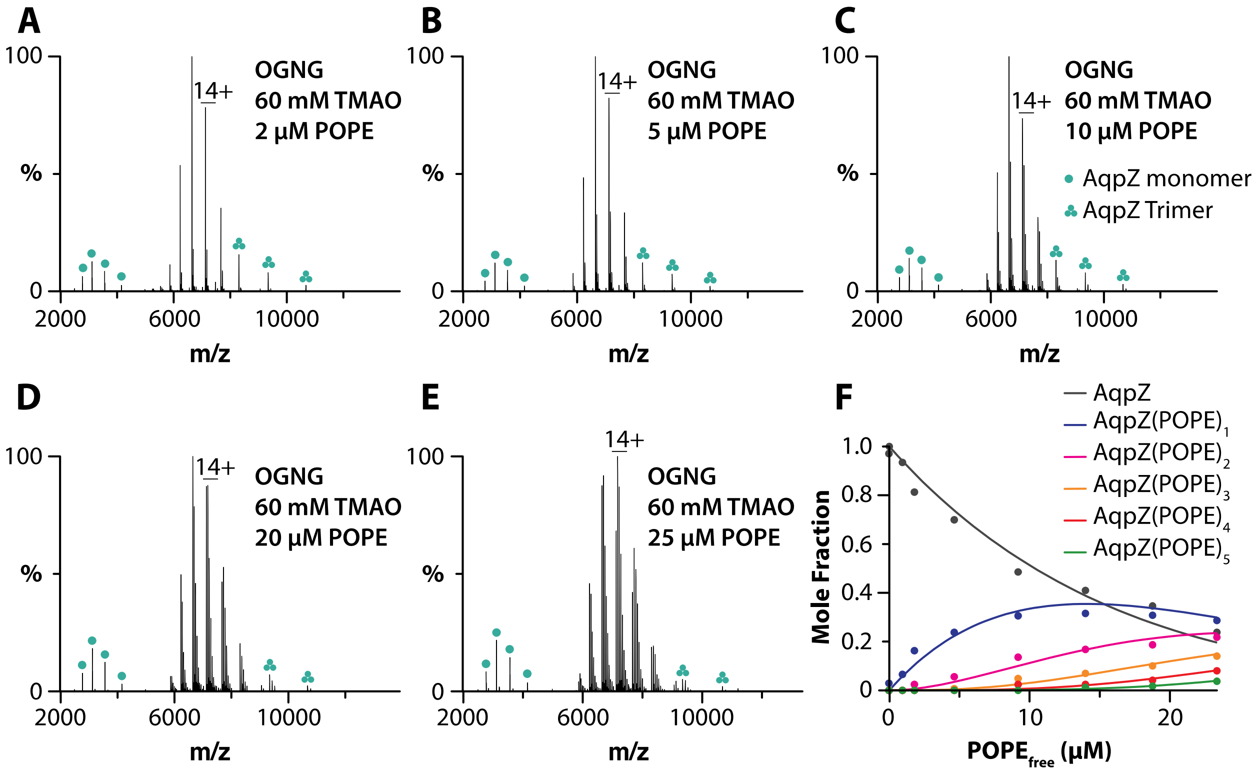

Figure S30. Determination of AqpZ-POPE equilibrium binding constants. A-E) AqpZ (1 µM) in OGNG and 60 mM TMAO mixed with different concentrations of POPE. F) Plot of mole fraction data (dots) determined from a titration series of POPE and subsequent fit of a sequential lipid binding model (lines).

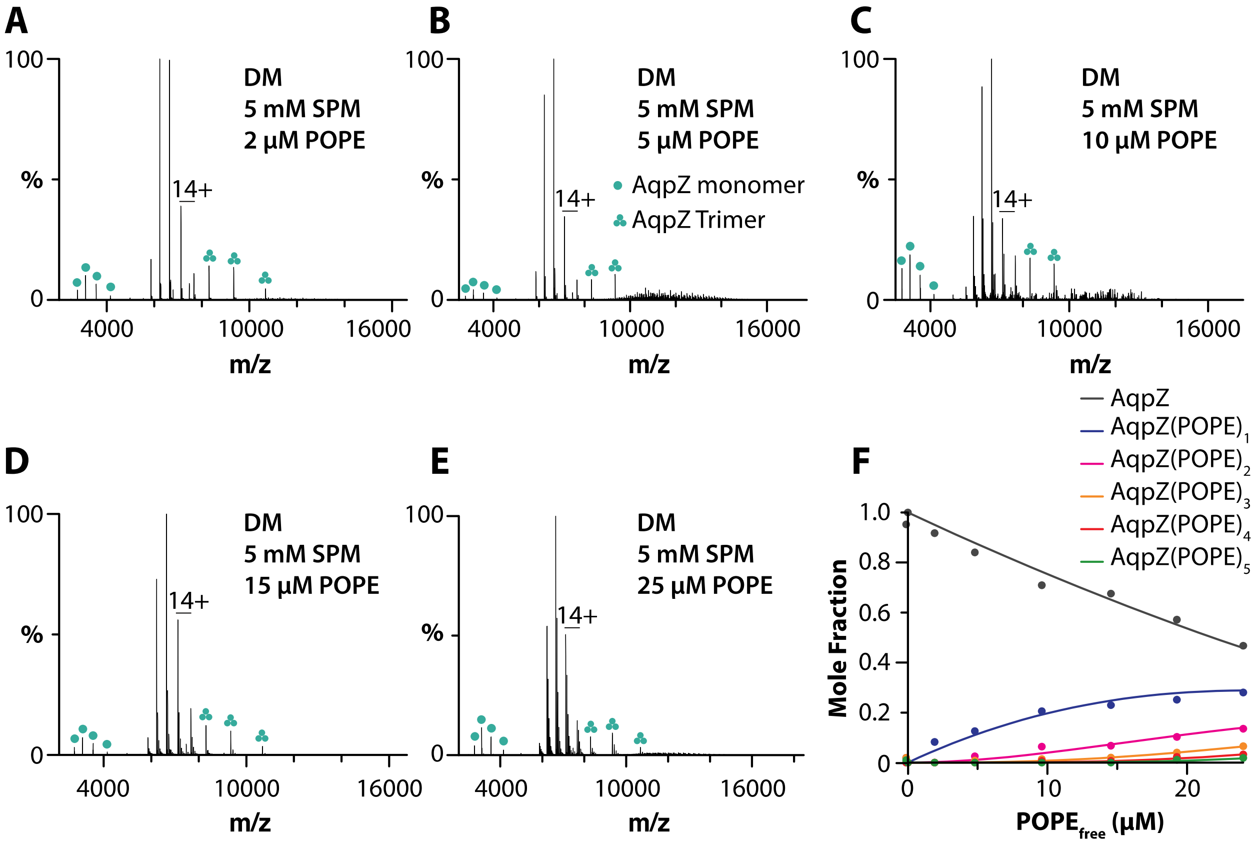

Figure S31. Determination of AqpZ-POPE equilibrium binding constants. A-E) AqpZ (1 µM) in DM and 5 mM SPM mixed with different concentrations of POPE. F) Plot of mole fraction data (dots) determined from a titration series of POPE and subsequent fit of a sequential lipid binding model (lines).

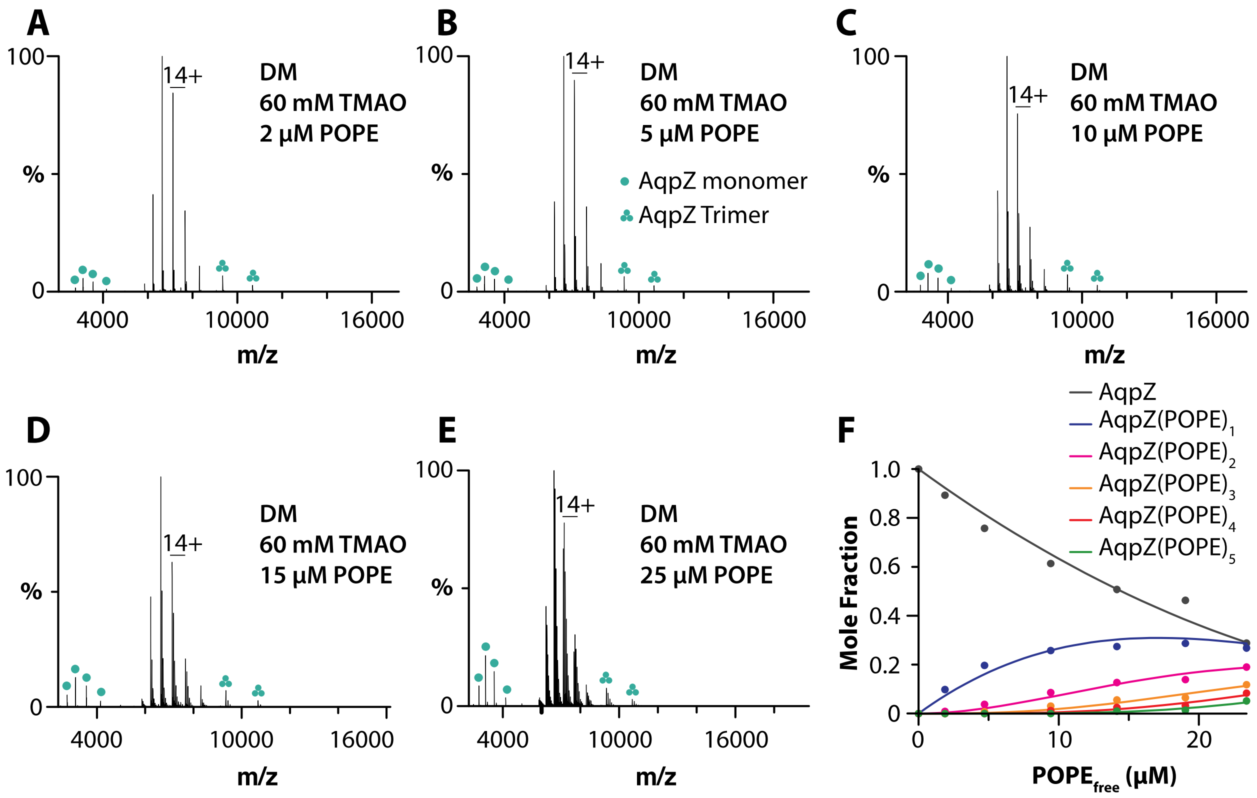

Figure S32. Determination of AqpZ-POPE equilibrium binding constants. A-E) AqpZ (1 μM) in DM and 60 mM TMAO mixed with different concentrations of POPE. F) Plot of mole fraction data (dots) determined from a titration series of POPE and subsequent fit of a sequential lipid binding model (lines).

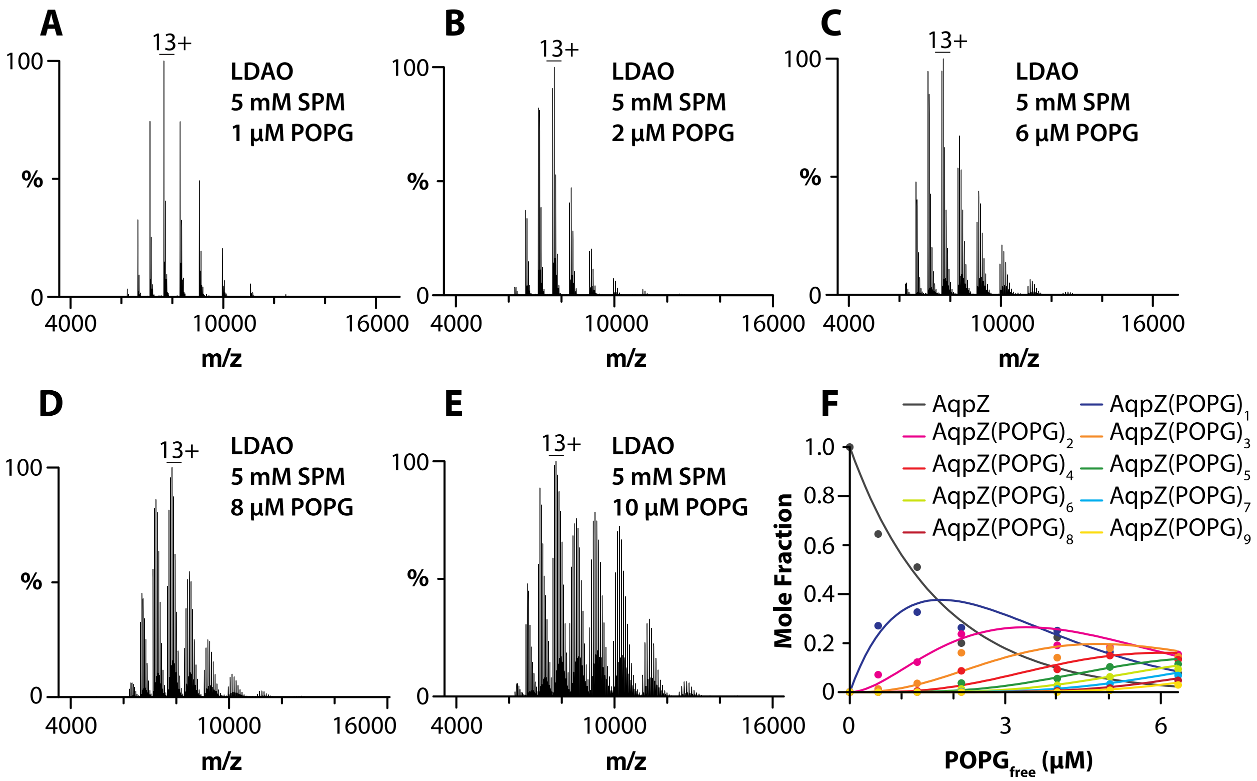

Figure S33. Determination of AqpZ-POPG equilibrium binding constants. A-E) AqpZ (1 μM) in LDAO and 5 mM SPM mixed with different concentrations of POPG. F) Plot of mole fraction data (dots) determined from a titration series of POPG and subsequent fit of a sequential lipid binding model (lines).

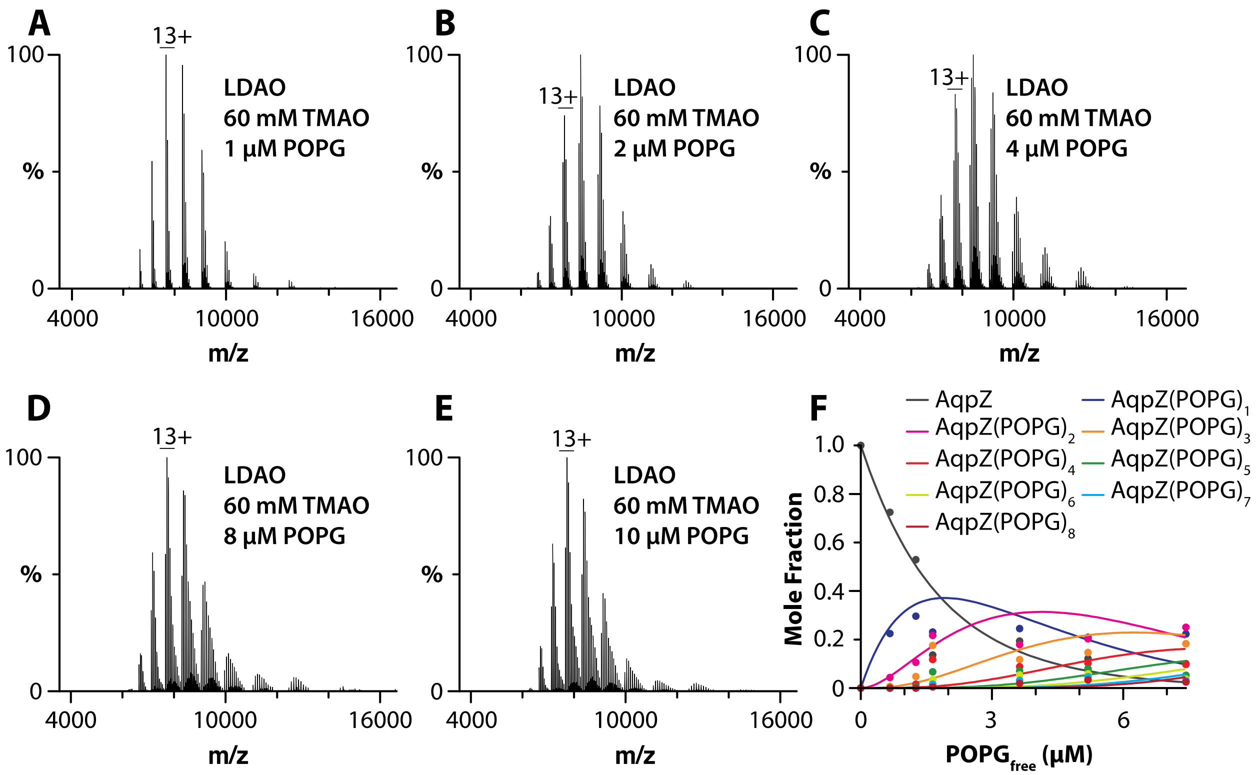

Figure S34. Determination of AqpZ-POPG equilibrium binding constants. A-E) AqpZ (1 μM) in LDAO and 60 mM TMAO mixed with different concentrations of POPG. F) Plot of mole fraction data (dots) determined from a titration series of POPG and subsequent fit of a sequential lipid binding model (lines).

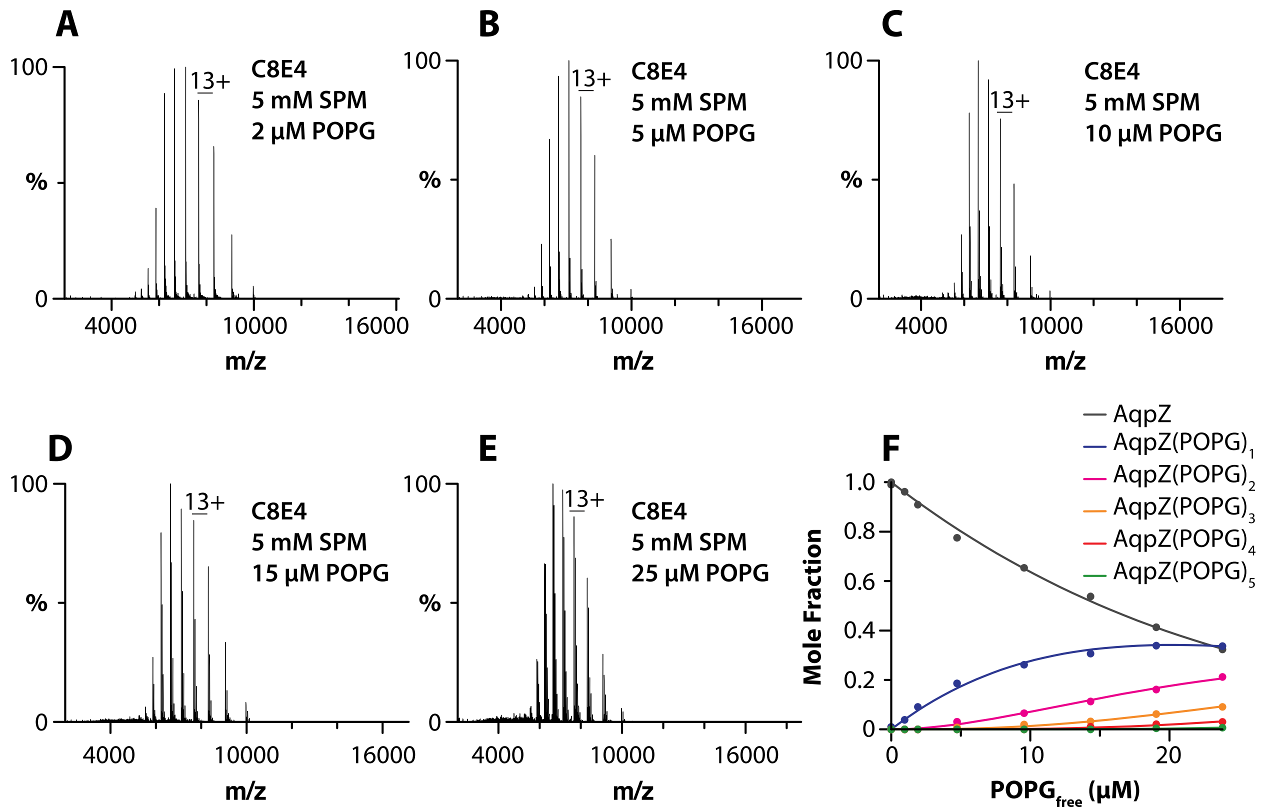

Figure S35. Determination of AqpZ-POPG equilibrium binding constants. A-E) AqpZ (1 µM) in C8E4 and 5 mM SPM mixed with different concentrations of POPG. F) Plot of mole fraction data (dots) determined from a titration series of POPG and subsequent fit of a sequential lipid binding model (lines).

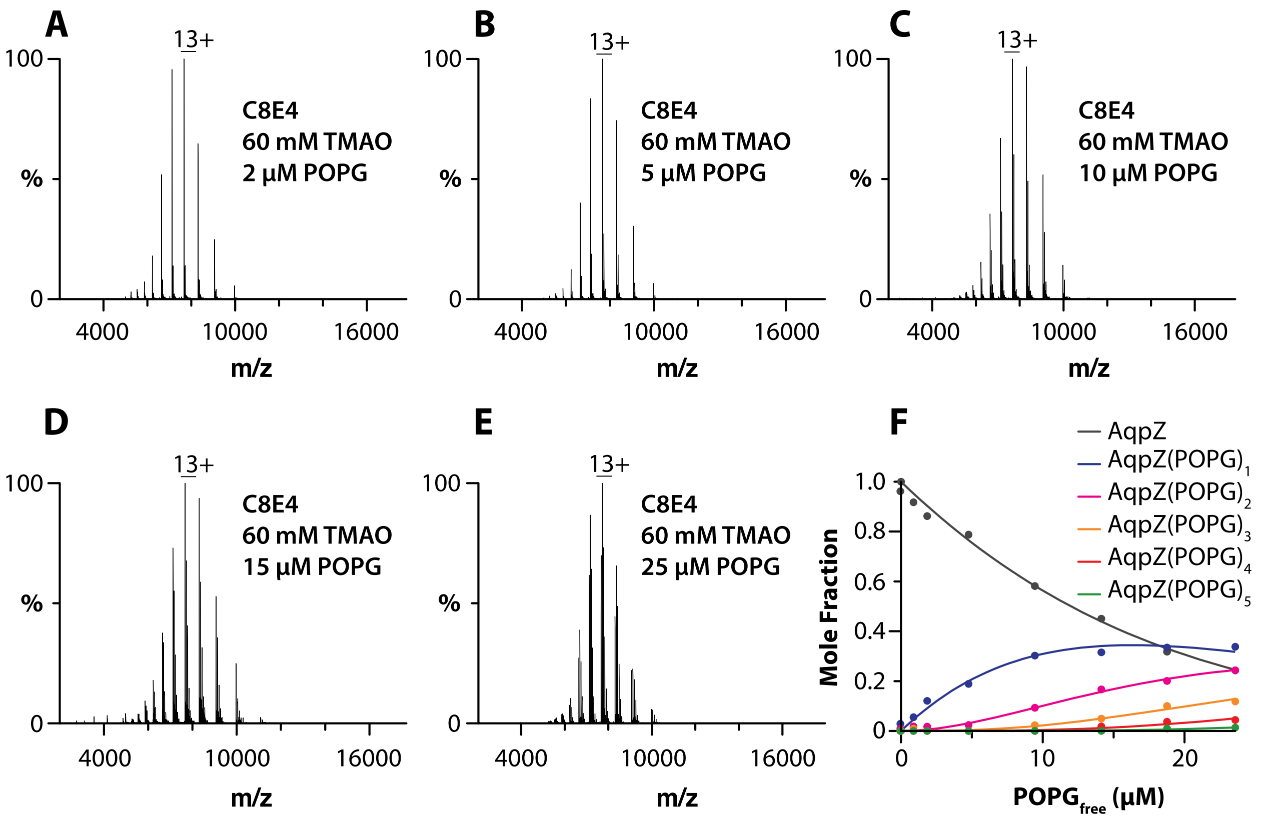

Figure S36. Determination of AqpZ-POPG equilibrium binding constants. A-E) AqpZ (1 µM) in C8E4 and 60 mM TMAO mixed with different concentrations of POPG. F) Plot of mole fraction data (dots) determined from a titration series of POPG and subsequent fit of a sequential lipid binding model (lines).

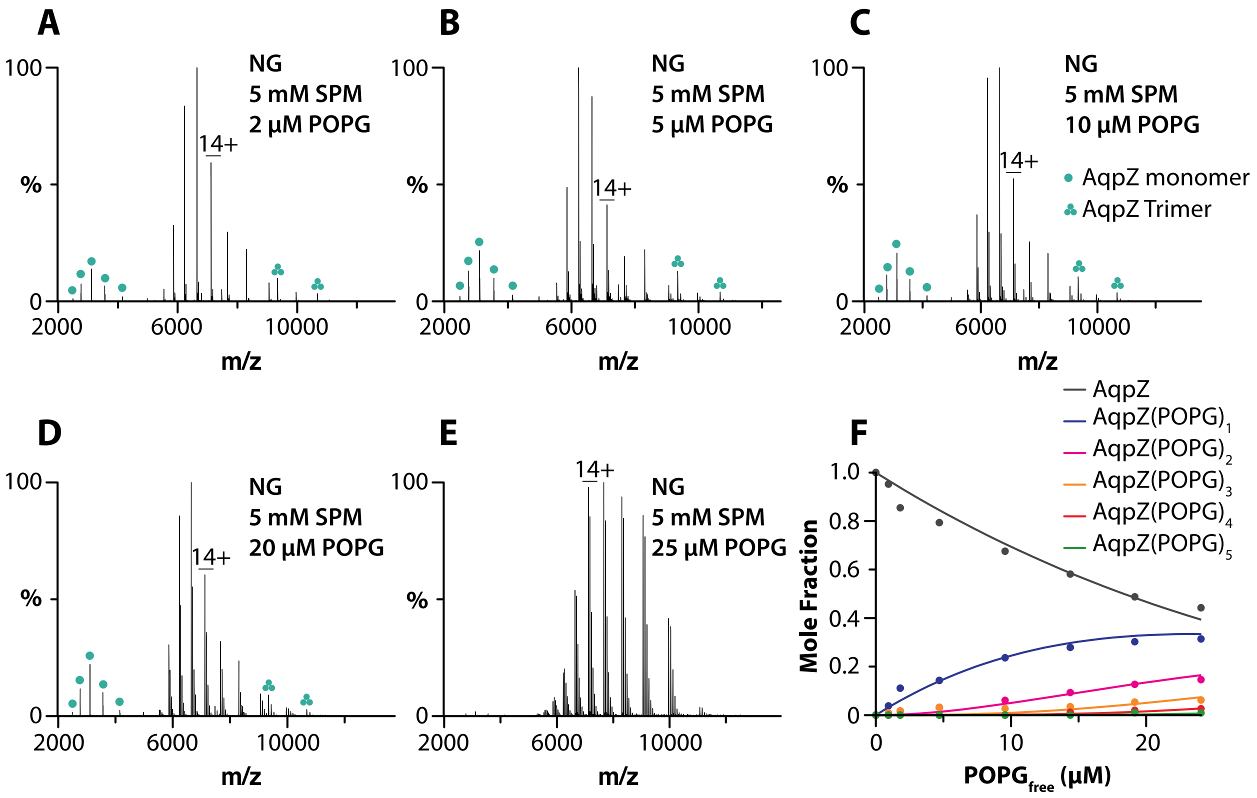

Figure S37. Determination of AqpZ-POPG equilibrium binding constants. A-E) AqpZ (1 μM) in NG and 5 mM SPM mixed with different concentrations of POPG. F) Plot of mole fraction data (dots) determined from a titration series of POPG and subsequent fit of a sequential lipid binding model (lines).

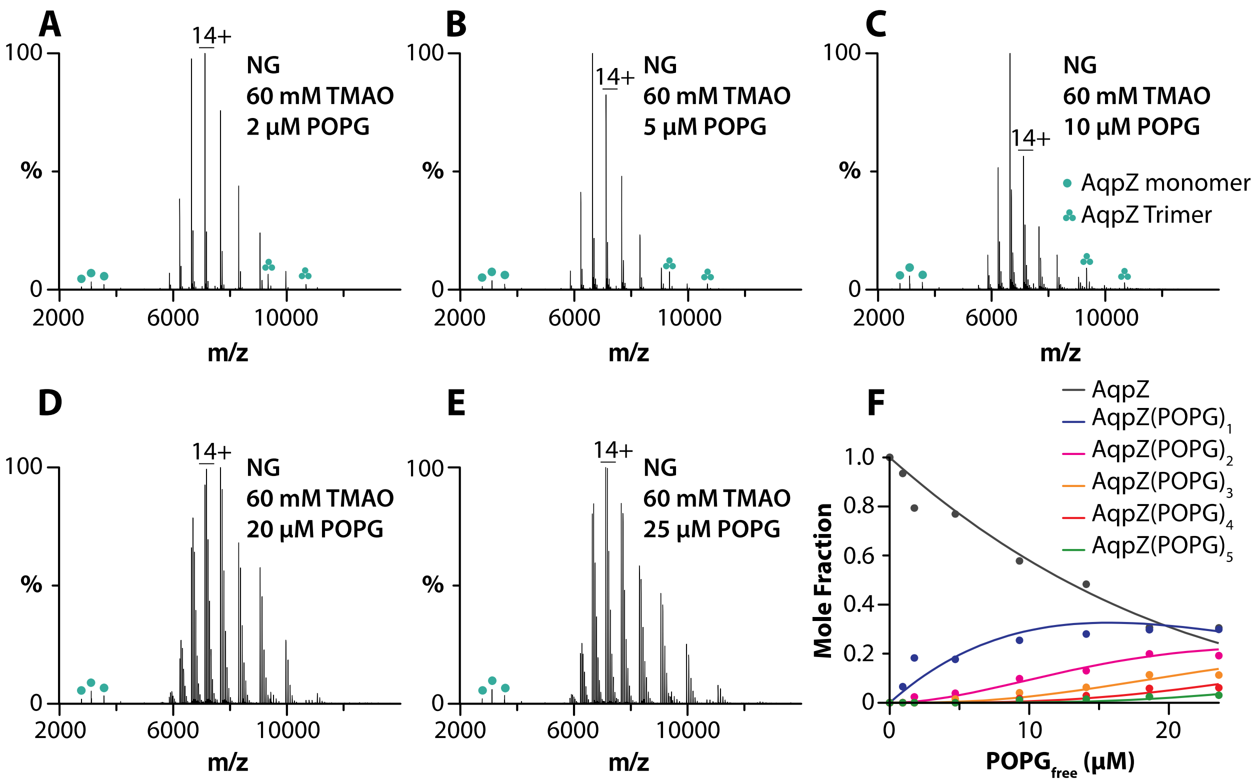

Figure S38. Determination of AqpZ-POPG equilibrium binding constants. A-E) AqpZ (1 µM) in NG and 60 mM TMAO mixed with different concentrations of POPG. F) Plot of mole fraction data (dots) determined from a titration series of POPG and subsequent fit of a sequential lipid binding model (lines).

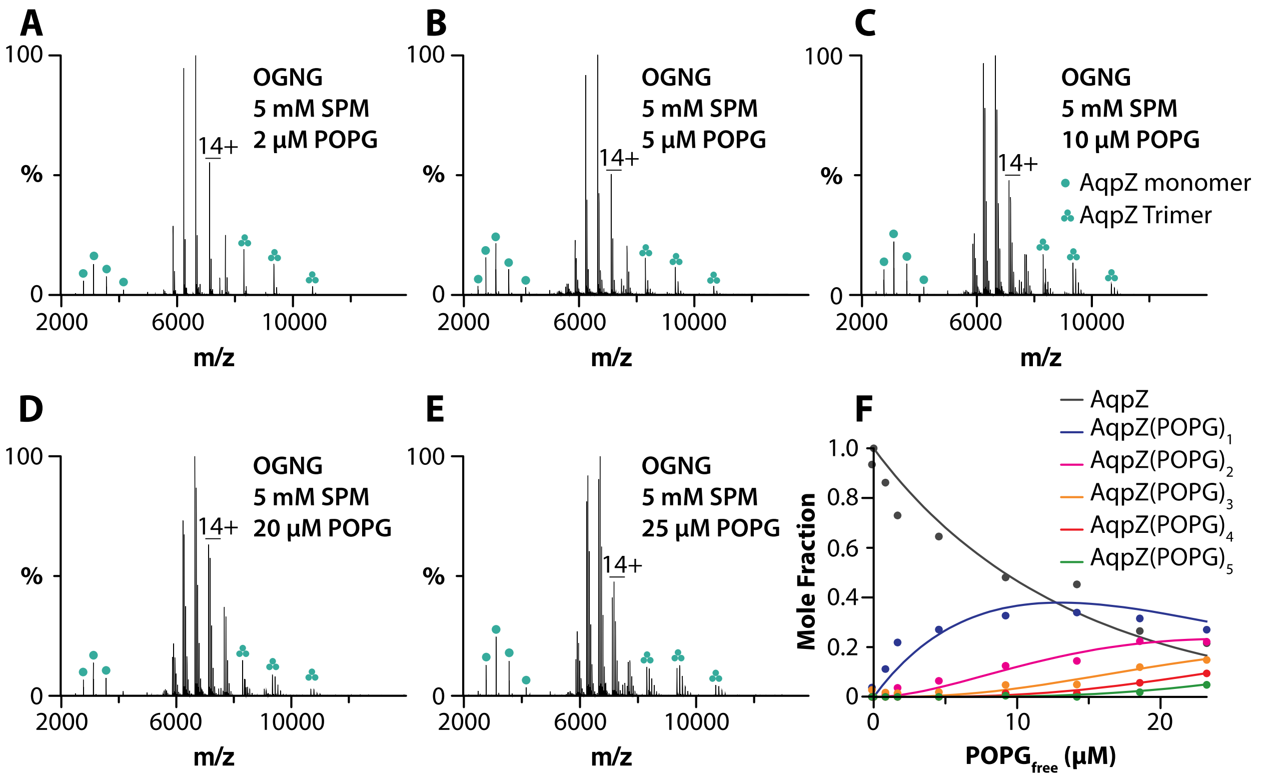

Figure S39. Determination of AqpZ-POPG equilibrium binding constants. A-E) AqpZ (1 μM) in OGNG and 5 mM SPM mixed with different concentrations of POPG. F) Plot of mole fraction data (dots) determined from a titration series of POPG and subsequent fit of a sequential lipid binding model (lines).

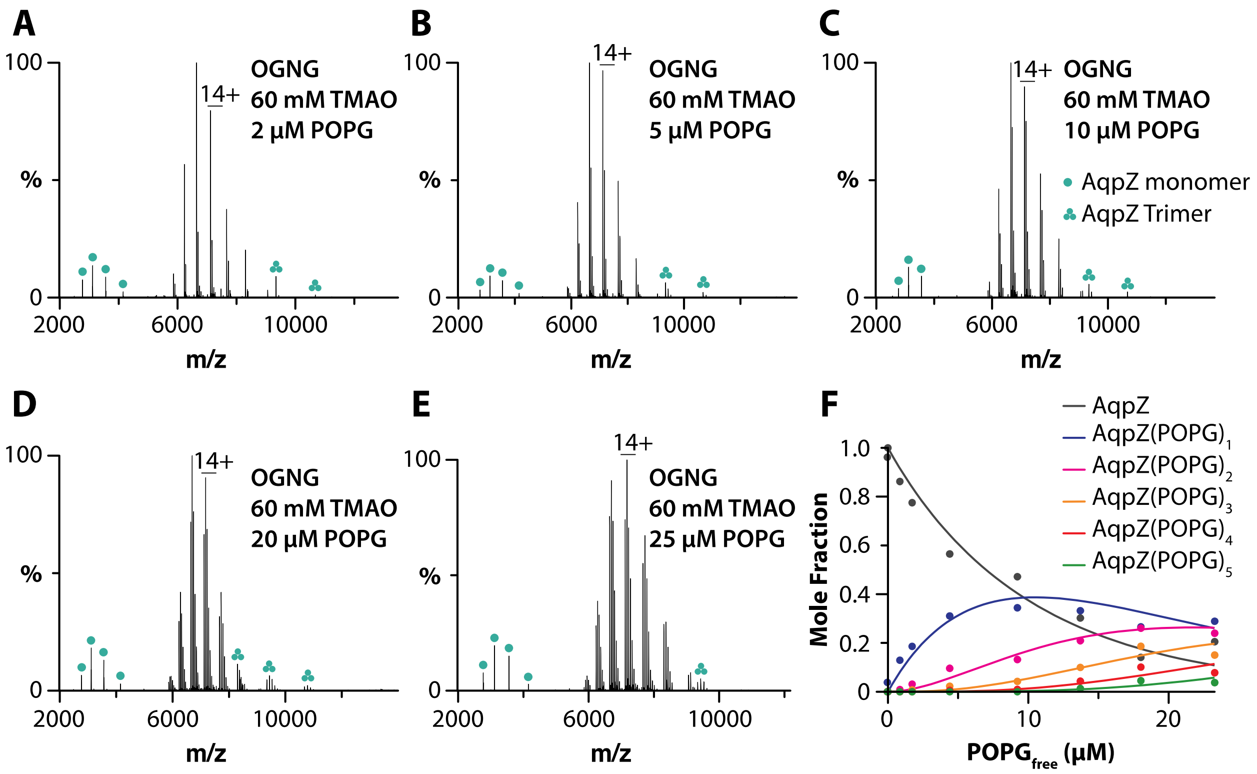

Figure S40. Determination of AqpZ-POPG equilibrium binding constants. A-E) AqpZ (1 μM) in OGNG and 60 mM TMAO mixed with different concentrations of POPG. F) Plot of mole fraction data (dots) determined from a titration series of POPG and subsequent fit of a sequential lipid binding model (lines).

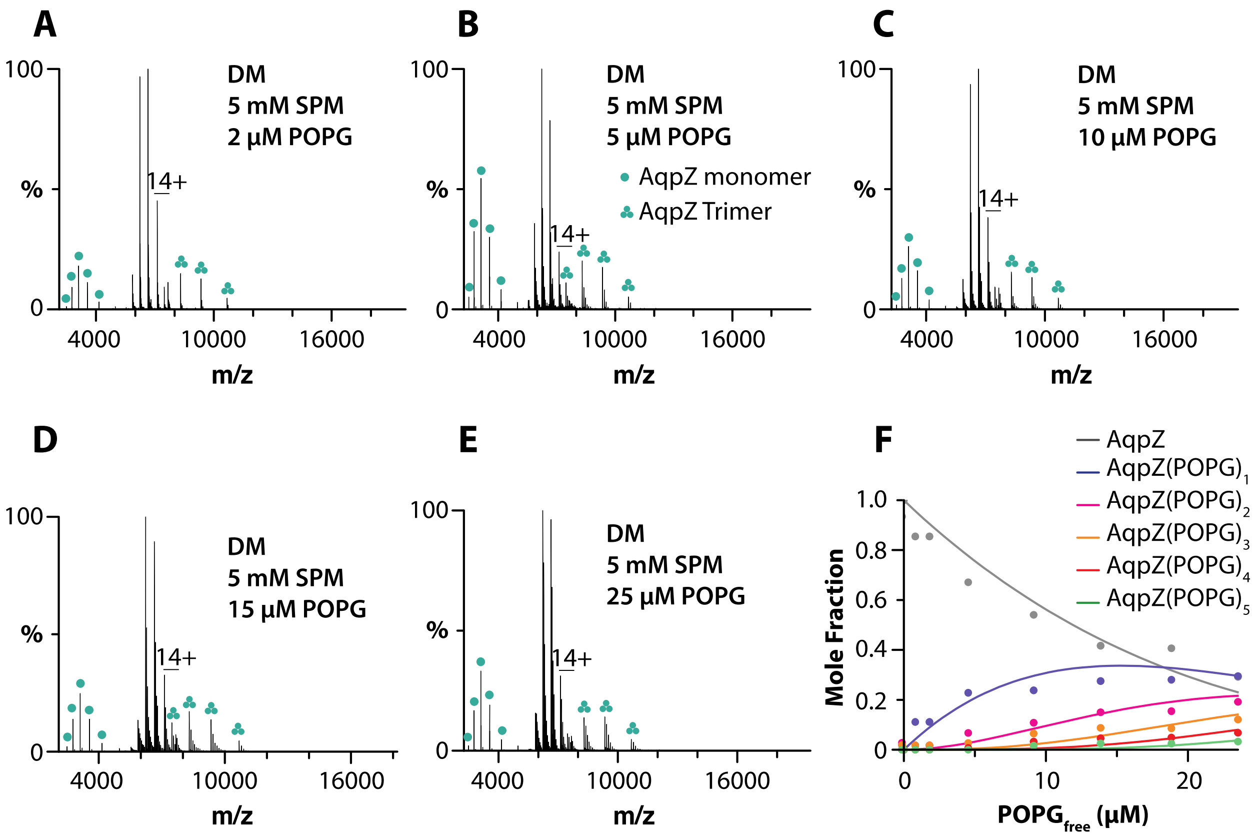

Figure S41. Determination of AqpZ-POPG equilibrium binding constants. A-E) AqpZ (1 µM) in DM and 5 mM SPM mixed with different concentrations of POPG. F) Plot of mole fraction data (dots) determined from a titration series of POPG and subsequent fit of a sequential lipid binding model (lines).

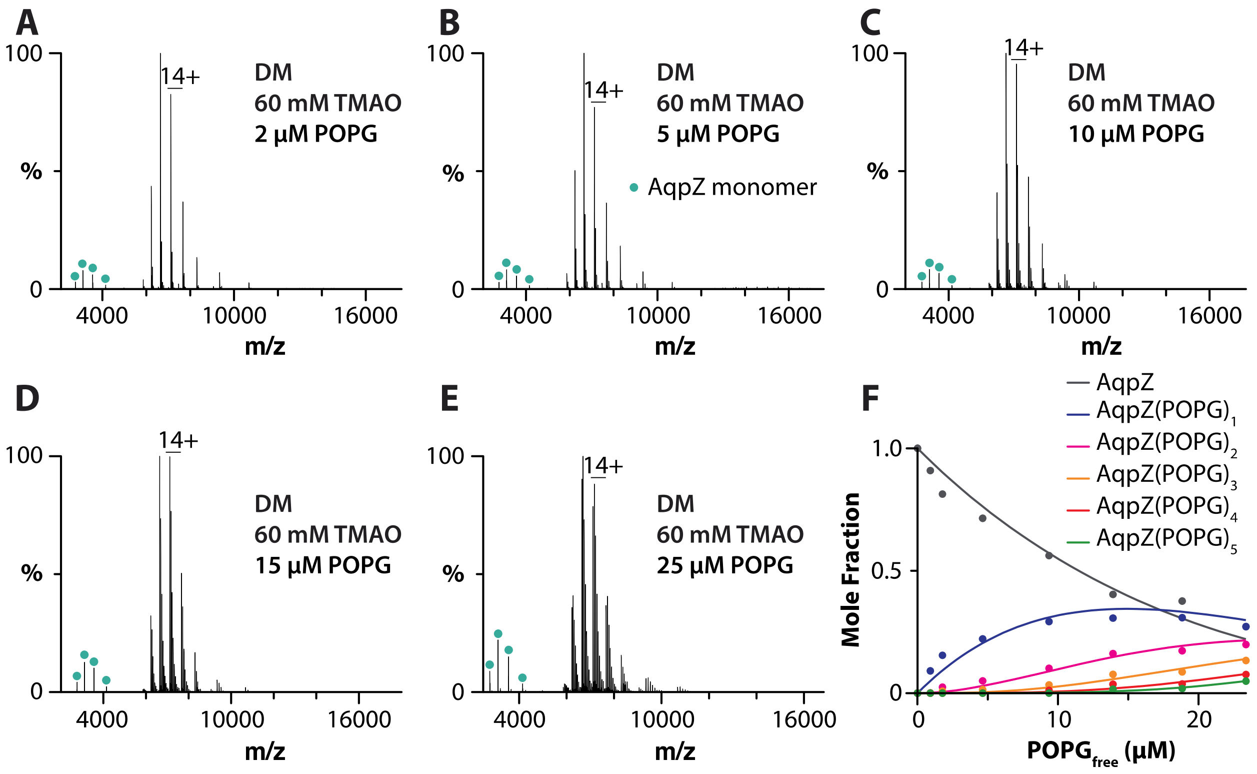

Figure S42. Determination of AqpZ-POPG equilibrium binding constants. A-E) AqpZ (1 μM) in DM and 60 mM TMAO mixed with different concentrations of POPG. F) Plot of mole fraction data (dots) determined from a titration series of POPG and subsequent fit of a sequential lipid binding model (lines).

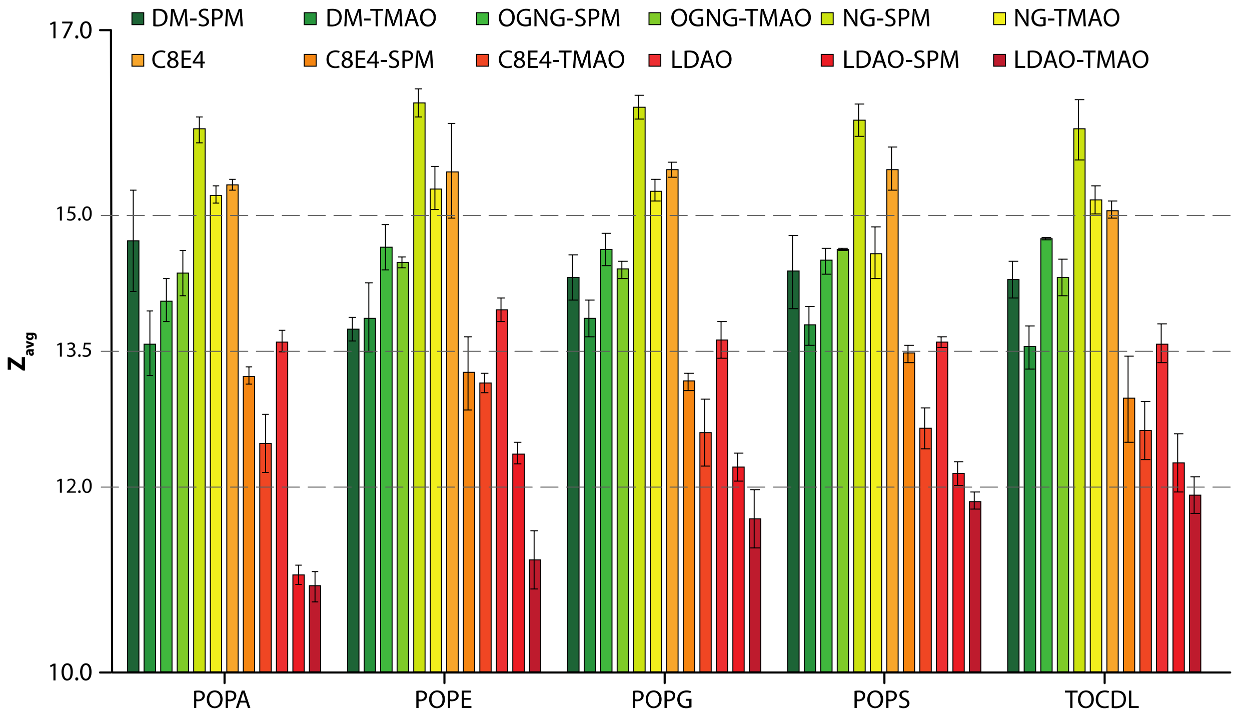

Figure S43. Z_{avg} for AqpZ in various detergents with different lipids. Reported are the mean and standard deviation (*n*=3).

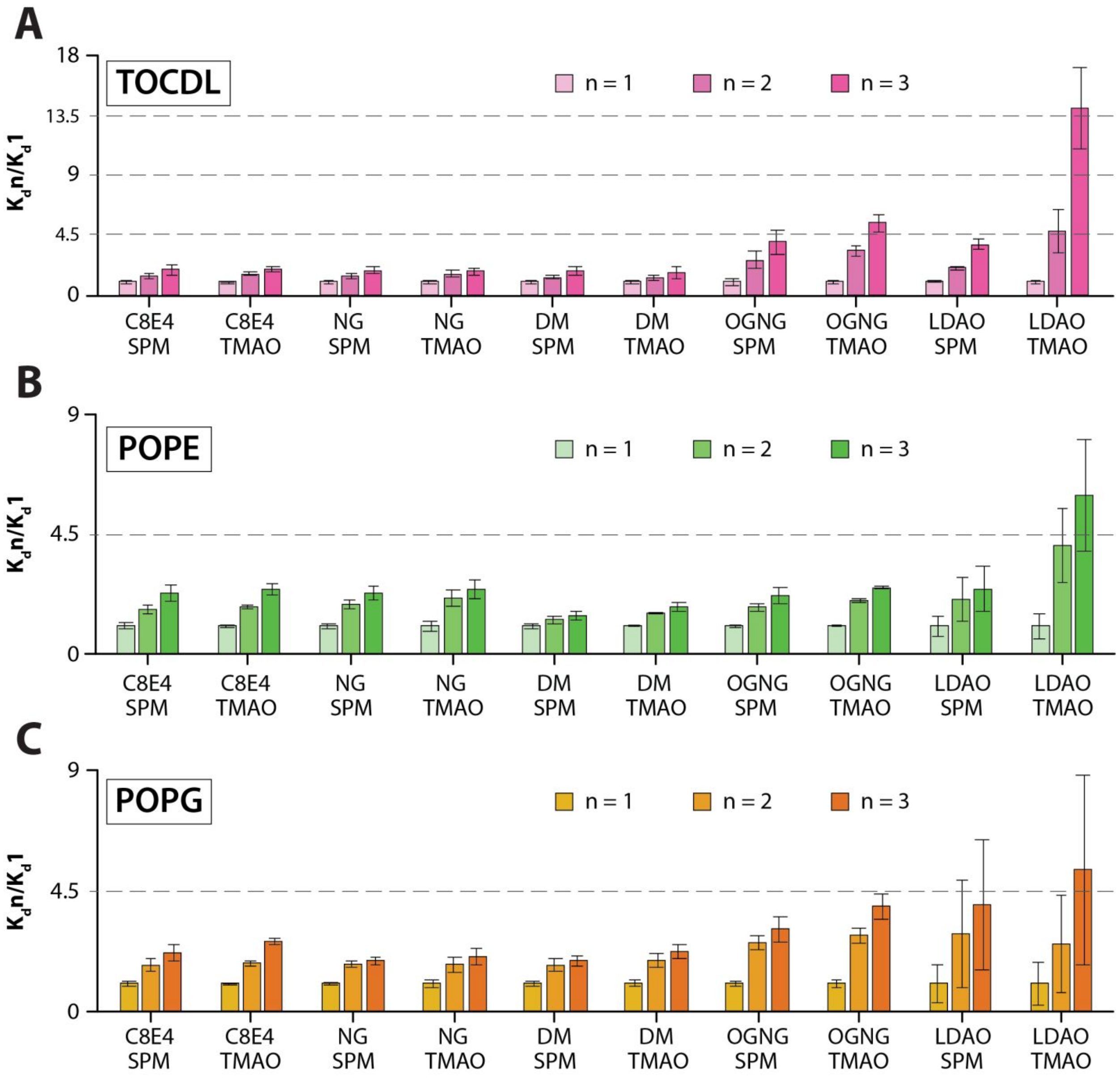

Figure S44. Fold change in K_ds for subsequent lipid binding to AqpZ in different detergents. Calculated for AqpZ in binding to A) TOCDL, B) POPE, and C) POPG. Reported are the mean and standard deviation (*n*=3).

Supporting Tables

Table S1. Instrument settings for analysis of AqpZ.

System	SID	HCD	Spray Voltage (kV)	Capillary Temperature (°C)	Trapping Pressure	Source DC Offset (V)	Injection Flatpole DC (V)	Inter Flatpole Lens (V)	Bent Flatpole DC (V)	Transfer Multipole DC (V)
**DM**	100	100	1.50	300	6	40.0	8.0	4.0	3.0	3.0
**DM-SPM**	100	80	1.60	300	6	25.0	8.0	5.0	16.0	3.0
**DM-TMAO**	100	80	1.60	300	6	25.0	8.0	5.0	16.0	3.0
**OGNG**	80	100	1.50	300	7	60.0	4.0	-20.0	10.0	6.0
**OGNG-SPM**	80	100	1.50	300	7	60.0	4.0	-20.0	10.0	6.0
**OGNG-TMAO**	80	100	1.50	300	7	60.0	4.0	-20.0	10.0	6.0
**NG**	60	70	1.70	200	5	25.0	8.0	7.0	6.0	2.0
**NG-SPM**	60	70	1.70	200	5	25.0	8.0	7.0	6.0	2.0
**NG-TMAO**	60	70	1.70	200	5	25.0	8.0	7.0	6.0	2.0
**C8E4**	60	70	1.70	200	5	25.0	8.0	7.0	6.0	2.0
**C8E4-SPM**	60	70	1.70	200	5	25.0	8.0	7.0	6.0	2.0
**C8E4-TMAO**	60	70	1.70	200	5	25.0	8.0	7.0	6.0	2.0
**LDAO**	60	70	1.70	200	5	25.0	8.0	7.0	6.0	2.0
**LDAO-SPM**	60	70	1.70	200	5	25.0	8.0	7.0	6.0	2.0
**LDAO-TMAO**	60	70	1.70	200	5	25.0	8.0	7.0	6.0	2.0

Table S2. Average charge state (Z_{avg}) of AqpZ in different detergent environments. Reported are mean and standard deviation (n=3).

System	Z_{avg}	# of charge states
**DM-SPM**	14.5 ± 0.1	4.0 ± 0.0
**DM-TMAO**	13.5 ± 0.4	4.7 ± 1.7
**OGNG**	17.4 ± 0.5	6.0 ± 0.0
**OGNG-SPM**	14.7 ± 0.2	5.7 ± 0.5
**OGNG-TMAO**	14.9 ± 0.4	4.0 ± 0.0
**NG**	18.8 ± 0.1	5.3 ± 0.5
**NG-SPM**	16.5 ± 0.2	7.7 ± 0.5
**NG-TMAO**	14.5 ± 0.3	6.3 ± 0.5
**C8E4**	15.3 ± 0.0	5.0 ± 0.0
**C8E4-SPM**	13.2 ± 0.1	6.7 ± 0.5
**C8E4-TMAO**	13.0 ± 0.2	5.7 ± 0.5
**LDAO**	13.5 ± 0.1	6.7 ± 0.9
**LDAO-SPM**	11.5 ± 0.0	7.3 ± 0.5
**LDAO-TMAO**	10.3 ± 0.2	6.3 ± 0.5

Table S3. Instrument settings for the analysis of AmtB-GlnK. Reported are mean and standard deviation (n=3).

System	SID	HCD	Spray Voltage (kV)	Capillary Temperature (°C)	Trapping Pressure	Source DC Offset (V)	Injection Flatpole DC (V)	Inter Flatpole Lens (V)	Bent Flatpole DC (V)	Transfer Multipole DC (V)
**DM**	50	90	1.60	200	6	30.0	8.0	5.0	16.0	3.0
**DM-SPM**	50	120	1.60	200	6	35.0	8.0	5.0	16.0	3.0
**DM-TMAO**	50	120	1.60	200	6	35.0	8.0	5.0	16.0	3.0
**OGNG**	80	100	1.50	300	7	60.0	4.0	-20.0	10.0	6.0
**OGNG-SPM**	80	100	1.50	300	7	60.0	4.0	-20.0	10.0	6.0
**OGNG-TMAO**	60	60	1.60	200	5	60.0	4.0	-20.0	10.0	6.0
**NG**	40	105	1.60	200	5	60.0	4.0	-20.0	10.0	6.0
**NG-SPM**	40	100	1.60	200	5	60.0	4.0	-20.0	10.0	6.0
**NG-TMAO**	70	80	1.60	200	5	60.0	4.0	-20.0	10.0	6.0
**C8E4**	60	60	1.60	200	5	60.0	4.0	-20.0	10.0	6.0
**C8E4-SPM**	60	60	1.60	200	5	60.0	4.0	-20.0	10.0	6.0
**C8E4-TMAO**	60	60	1.60	200	5	60.0	4.0	-20.0	10.0	6.0
**LDAO**	60	60	1.60	200	5	60.0	4.0	-20.0	10.0	6.0
**LDAO-SPM**	60	60	1.60	200	5	60.0	4.0	-20.0	10.0	6.0
**LDAO-TMAO**	60	60	1.60	200	5	60.0	4.0	-20.0	10.0	6.0

Table S4. Z_{avg} of AmtB-GlnK in different detergent environments.

System	Z_{avg}	# of Zs
**DM-SPM**	21.9 ± 0.2	5.7 ± 0.5
**NG**	25.8 ± 0.2	5.0 ± 0.0
**NG-SPM**	22.6 ± 0.3	6.7 ± 0.5
**C8E4**	20.5 ± 0.1	4.0 ± 0.0
**C8E4-SPM**	18.8 ± 0.4	6.0 ± 0.8
**C8E4-TMAO**	17.3 ± 0.3	5.0 ± 0.0
**LDAO**	20.0 ± 0.3	8.7 ± 0.5
**LDAO-SPM**	17.7 ± 0.5	7.3 ± 0.5

Table S5. Equilibrium dissociation constants (K_ds) for AqpZ-TOCDL in different detergents. Reported are the mean and standard deviation (*n* = 3)

Environment	K_d1 (µM)	K_d2 (µM)	K_d3 (µM)
**C8E4+5 mM SPM**	30.3 ± 2.7	45.3 ± 4.0	58.3 ± 11.2
**C8E4+60 mM TMAO**	22.8 ± 0.9	37.0 ± 1.6	45.9 ± 4.0
**NG+5 mM SPM**	28.8 ± 3.3	42.3 ± 2.5	54.4 ± 4.6
**NG+60 mM TMAO**	19.7 ± 1.6	31.5 ± 4.1	35.5 ± 4.9
**DM+5 mM SPM**	21.7 ± 2.1	30.0 ± 2.8	39.5 ± 5.6
**DM+60 mM TMAO**	14.1 ± 1.8	18.9 ± 2.2	23.8 ± 5.1
**OGNG+5 mM SPM**	7.8 ± 1.7	20.9 ± 1.3	31.1 ± 0.9
**OGNG+60 mM TMAO**	4.4 ± 0.5	14.9 ± 0.6	23.9 ± 0.2
**LDAO+5 mM SPM**	1.4 ± 0.1	2.8 ± 0.1	5.2 ± 0.4
**LDAO+60 mM TMAO**	0.3 ± 0.0	1.6 ± 0.5	4.7 ± 0.9

Table S6. K_ds for POPE binding to AqpZ in different detergents. Reported are the mean and standard deviation (n = 3)

Environment	K_d1 (μM)	K_d2 (μM)	K_d3 (μM)
**C8E4+5 mM SPM**	28.2 ± 2.3	46.1 ± 2.4	63.2 ± 7.0
**C8E4+60 mM TMAO**	23.0 ± 0.5	39.3 ± 1.3	55.0 ± 5.0
**NG+5 mM SPM**	44.3 ± 3.0	80.2 ± 5.4	99.5 ± 9.7
**NG+60 mM TMAO**	30.7 ± 3.8	63.0 ± 5.2	73.4 ± 5.3
**DM+5 mM SPM**	37.8 ± 2.4	47.1 ± 4.5	52.4 ± 4.9
**DM+60 mM TMAO**	23.7 ± 0.3	35.3 ± 0.9	41.1 ± 3.6
**OGNG+5 mM SPM**	19.7 ± 0.7	33.6 ± 2.8	42.3 ± 6.1
**OGNG+60 mM TMAO**	15.1 ± 0.3	29.6 ± 0.7	37.0 ± 0.0
**LDAO+5 mM SPM**	4.2 ± 1.1	8.5 ± 2.5	10.1 ± 2.3
**LDAO+60 mM TMAO**	1.3 ± 0.4	5.2 ± 0.4	7.6 ± 0.7

Table S7. K_ds for AqpZ-POPG in different detergents. Reported are the mean and standard deviation (*n* = 3)

Environment	K_d1 (μM)	K_d2 (μM)	K_d3 (μM)
**C8E4+5 mM SPM**	25.8 ± 2.0	43.5 ± 4.9	55.5 ± 6.9
**C8E4+60 mM TMAO**	18.4 ± 0.1	32.2 ± 1.8	47.3 ± 2.3
**NG+5 mM SPM**	27.0 ± 1.2	46.3 ± 2.9	49.8 ± 2.7
**NG+60 mM TMAO**	19.0 ± 1.8	32.4 ± 4.6	38.3 ± 4.7
**DM+5 mM SPM**	19.9 ± 1.2	33.7 ± 4.0	36.8 ± 2.8
**DM+60 mM TMAO**	15.5 ± 1.3	28.8 ± 3.1	34.0 ± 2.5
**OGNG+5 mM SPM**	12.0 ± 0.6	30.1 ± 2.5	36.0 ± 5.1
**OGNG+60 mM TMAO**	8.8 ± 0.8	24.5 ± 1.1	34.0 ± 2.6
**LDAO+5 mM SPM**	2.0 ± 1.0	5.6 ± 2.8	7.8 ± 2.8
**LDAO+60 mM TMAO**	1.2 ± 0.7	2.8 ± 1.3	6.0 ± 2.1

Table S8. CMC of different detergents used in this study.

Detergent	CMC (wt/v%)	CMC (mM)
**DM** (n-decyl-ß-maltoside)	0.087	~1.8
**OGNG** (octyl glucose neopentyl glycol)	0.058	~1.02
**NG** (n-nonyl-ß-D-glucopyranoside)	0.2	~6.5
**C8E4** (tetraethylene glycol monooctyl ether)	0.25	~8
**LDAO** (lauryl dimethylamine N-oxide)	0.023	~1-2

References

(1) Laganowsky, A.; Reading, E.; Allison, T. M.; Ulmschneider, M. B.; Degiacomi, M. T.; Baldwin, A. J.; Robinson, C. V. Membrane proteins bind lipids selectively to modulate their structure and function. *Nature* **2014**, *510* (7503), 172-175. DOI: 10.1038/nature13419.
(2) Laganowsky, A.; Reading, E.; Hopper, J. T.; Robinson, C. V. Mass spectrometry of intact membrane protein complexes. *Nat Protoc* **2013**, *8* (4), 639-651. DOI: 10.1038/nprot.2013.024.
(3) Cong, X.; Liu, Y.; Liu, W.; Liang, X.; Laganowsky, A. Allosteric modulation of protein-protein interactions by individual lipid binding events. *Nat Commun* **2017**, *8* (1), 2203. DOI: 10.1038/s41467-017-02397-0.
(4) Cong, X.; Liu, Y.; Liu, W.; Liang, X.; Russell, D. H.; Laganowsky, A. Determining Membrane Protein–Lipid Binding Thermodynamics Using Native Mass Spectrometry. *Journal of the American Chemical Society* **2016**, *138* (13), 4346-4349. DOI: 10.1021/jacs.6b01771.
(5) Marty, M. T.; Baldwin, A. J.; Marklund, E. G.; Hochberg, G. K.; Benesch, J. L.; Robinson, C. V. Bayesian deconvolution of mass and ion mobility spectra: from binary interactions to polydisperse ensembles. *Anal Chem* **2015**, *87* (8), 4370-4376. DOI: 10.1021/acs.analchem.5b00140.
